# Survival for waitlisted kidney failure patients receiving transplantation versus remaining on waiting list: systematic review and meta-analysis

**DOI:** 10.1136/bmj-2021-068769

**Published:** 2022-03-01

**Authors:** Daoud Chaudhry, Abdullah Chaudhry, Javeria Peracha, Adnan Sharif

**Affiliations:** 1 School of Medical and Dental Sciences, University of Birmingham, Birmingham, UK; 2 Department of Nephrology and Transplantation, Queen Elizabeth Hospital, Edgbaston, Birmingham, UK; 3 Institute of Immunology and Immunotherapy, University of Birmingham, Birmingham, UK

## Abstract

**Objectives:**

To investigate the survival benefit of transplantation versus dialysis for waitlisted kidney failure patients with a priori stratification.

**Design:**

Systematic review and meta-analysis.

**Data sources:**

Online databases MEDLINE, Ovid Embase, Web of Science, Cochrane Collection, and ClinicalTrials.gov were searched between database inception and 1 March 2021.

**Inclusion criteria:**

All comparative studies that assessed all cause mortality for transplantation versus dialysis in patients with kidney failure waitlisted for transplant surgery were included. Two independent reviewers extracted the data and assessed the risk of bias of included studies. Meta-analysis was done using the DerSimonian-Laird random effects model, with heterogeneity investigated by subgroup analyses, sensitivity analyses, and meta-regression.

**Results:**

The search identified 48 observational studies with no randomised controlled trials (n=1 245 850 patients). In total, 92% (n=44/48) of studies reported a long term (at least one year) survival benefit associated with transplantation compared with dialysis. However, 11 of those studies identified stratums in which transplantation offered no statistically significant benefit over remaining on dialysis. In 18 studies suitable for meta-analysis, kidney transplantation showed a survival benefit (hazard ratio 0.45, 95% confidence interval 0.39 to 0.54; P<0.001), with significant heterogeneity even after subgroup/sensitivity analyses or meta-regression analysis.

**Conclusion:**

Kidney transplantation remains the superior treatment modality for most patients with kidney failure to reduce all cause mortality, but some subgroups may lack a survival benefit. Given the continued scarcity of donor organs, further evidence is needed to better inform decision making for patients with kidney failure.

**Study registration:**

PROSPERO CRD42021247247.

## Introduction

Transplantation is established as the gold standard therapy of choice for patients with kidney failure because of improvements in mortality and quality of life for most eligible candidates. However, not all patients with kidney failure are deemed eligible for kidney transplant surgery owing to their personalised risk-benefit determination. For example, the latest UK Renal Registry report (up to 31 December 2019) shows that 28 303 patients with kidney failure are managed on haemodialysis or peritoneal dialysis,^1^ with a greater but undefined number of patients living with advanced chronic kidney disease. Yet as of 31 March 2021, only 3525 patients were actively awaiting kidney transplant surgery (with 4321 waitlisted but suspended).^2^ This suggests that not every patient with kidney failure is deemed suitable for the rigours of general anaesthesia, kidney transplant surgery, and/or complications associated with long term need for immunosuppression.

Although many studies have published their individual findings, Tonelli and colleagues were the last, a decade ago, to do a systematic review of cohort studies comparing adult kidney transplant recipients against patients on chronic dialysis.^3^ From 110 eligible studies, including 1 922 300 participants from 27 different countries, they found a significantly lower mortality risk associated with kidney transplantation and an increase in the relative magnitude of the survival benefit over time (P<0.001). However, patient and/or study level factors associated with greater or lesser benefit from transplantation were not identified. Additionally, because of the broad approach, most of the cohort studies included compared transplantation with all patients on dialysis, which introduces a significant selection bias.

Although many patients with kidney failure have contraindications prohibiting them from being considered as candidates for kidney transplantation, comparing survival of waitlisted patients with kidney failure who proceed with transplantation versus those who remain on dialysis allows a meaningful comparison. In a subset of studies (n=10), which compared transplant recipients with waitlisted patients on dialysis, Tonelli and colleagues found that the benefits of transplantation remained significant but were less pronounced.^3^ Moreover, although studies from across the globe were included, data were not stratified on the basis of geographical region, meaning that concerns about external validity remained.

In the context of continued disparity in the supply of and demand for donors, understanding which subgroups of patients with kidney failure may not attain survival benefits after kidney transplantation is important for counselling and clinical decision making. Any recommendations about risk versus benefit of kidney transplant surgery for eligible kidney transplant candidates should be based on the best available evidence. Evidence has been gained across several cohorts, but a comprehensive and contemporary review using recommended analytical techniques is lacking. We, therefore, systematically reviewed the survival benefit of kidney transplantation versus remaining on dialysis for waitlisted patients with kidney failure, with a meta-analysis of studies reporting eligible empirical data.

## Methods

The study protocol was prospectively registered on the PROSPERO database (CRD42021247247) and conducted in accordance with PRISMA (Preferred Reporting Items for Systematic Reviews and Meta-Analyses).^4^

### Eligibility criteria

We included all studies comparing mortality between patients with kidney failure deemed suitable for transplant surgery (that is, waitlisted) receiving transplantation versus those remaining on dialysis with minimum one year of follow-up. We excluded studies based solely on paediatric populations (age <16 years) or multi-organ transplant recipients. We included only studies of primary kidney transplantation, as re-transplants represent a small fraction of transplant candidates with a different risk “phenotype” with regards to survival outcomes.^5^ ^6^ We also excluded studies with small cohort size (<30 patients), as Tonelli and colleagues did, to optimise work without appreciable loss of power and inflation of bias. We excluded reviews, expert opinions, editorials, correspondences, and case reports.

For eligibility criteria of the meta-analysis, we excluded studies reporting data before the 1980s as, although informative for a narrative synthesis, they do not reflect current practice for pooled analysis. We also reviewed studies for data source, time period, population structure, and geographical region. Where studies presented overlapping populations, with respect to time periods and geographical regions, we gave priority for study selection to the more contemporary or largest data cohort. Similarly, in the context of two competing studies with overlapping patient cohorts (for example, age), we gave selection priority to the study with a general patient cohort (for example, all adults) over specific patient cohorts (for example, older adults only). No two studies with potential overlapping patient data could be selected together.

### Search strategy

Two reviewers (DC and AC) independently searched the following databases: MEDLINE, Ovid Embase, Web of Science, Cochrane Collection, and ClinicalTrials.gov. Searches were performed from inception to 1 March 2021, using a diverse amalgamation of Medical Subject Heading (MeSH) terms and search terms tailored to the tree structure and descriptors of each database to improve their reach (see supplementary table A). We placed no limits on language or year of publication.

### Selection of studies

Two reviewers (DC and AC) independently screened all records retrieved from the databases for relevancy on the hierarchical basis of title, abstract, and finally full text review for eligible studies. For studies reported in a non-English language, we sought appropriate translators before assessment. Any disagreements were resolved through discussion or arbitration with a third reviewer (AS). We followed up studies without accessible abstracts or full text by contacting the British Library (through interlibrary loan), along with the corresponding primary authors of the study. After failing to gain accessibility after these steps, we excluded studies. After full text analysis, we screened reference lists of all eligible publications to flag additional studies.

### Data extraction and outcome measures

The two reviewers (DC and AC) extracted data independently from eligible studies, with consultation with a third reviewer (AS) as needed. We collected extracted data by using a standardised piloted data collection spreadsheet (Excel, Microsoft Corp, WA, US). Data extracted included study characteristics (study identifier, year of publication, country, source of funding, data source, period of inclusion, follow-up duration, statistical approach adopted for survival analysis, sample size of waitlisted and transplanted patients, and special subgroups of population), population characteristics (age, sex, ethnicity, cause of primary kidney disease, and comorbidities), treatment characteristics (dialysis type, donor type, and immunosuppressive therapy regimen), and outcomes. The main outcome measure was all cause mortality. Owing to heterogeneity highlighted during scoping searches, we deemed all forms of patient survival data acceptable, including but not limited to unadjusted hazard ratios or risk ratios, adjusted hazard ratios or risk ratios, survival curves, and crude dichotomous event rates. Adjusted hazard ratios and their respective confidence intervals were the primary outcome measures of interest for the meta-analysis; we noted and summarised the remaining measures to aid narrative synthesis. Where studies reported subgroup data for different comorbidities, age groups, or donor types, we extracted data for each stratum.

### Risk of bias quality scoring

Two reviewers (DC and AC) assessed the quality of studies and their risk of bias independently by using the Newcastle-Ottawa Assessment Scale (NOS) for comparative non-randomised studies (cohort or case-control).^7^ ^8^ The NOS consists of three quality parameters: selection of study participants (4 stars), quality of adjustment for confounding (2 stars), and ascertainment of the exposure or outcome of interest (3 stars). For the confounding criteria, 1 star was allocated if groups were comparable on variables of age and sex, and an additional star if groups were comparable on cause of primary renal disease and comorbidity burden. Therefore, the maximum available score was 9, representing the highest methodological quality. Despite lacking any formalised criteria for what score constitutes a “high quality study,” many papers have conventionally regarded a NOS score ≥7 as the threshold.^9^ For this study, we adopted a more stringent approach, with a total score of ≤5 considered low, 6-7 considered moderate, and 8-9 deemed high quality. Any discrepancies between reviewers were resolved by discussion or arbitration with a third reviewer (AS).

### Data synthesis and statistical analysis

We summarised the results of the systematic review both qualitatively and quantitatively. In the absence of individual patient data, we followed the recommendations of the Cochrane Handbook for Systematic Reviews of Interventions and did the meta-analysis of time-to-event data by pooling reported long term mortality hazard ratios from studies.^10^

Where possible, we chose the most adjusted overall estimate of transplantation versus dialysis (that is, multivariate regression over univariate regression). If a study reported regression estimates only by subgroup (for example, by donor type), we entered estimates separately into the analysis. If studies reported Cox regression results as relative risks, we regarded them as hazard ratios. When hazard ratios and their 95% confidence intervals were unavailable, we estimated the hazard ratios, provided sufficient information was present (for example, log-rank test P values or Kaplan-Meier curves), using the statistical procedures described by Parmar et al in 1998 and Tierney et al. in 2007.^11^ ^12^ To aid in this later step, Kaplan-Meier curves were digitalised with Digitizelt.^13^

After calculation of the natural logarithmic hazard ratio and its standard error for each viable study, was used the DerSimonian-Laird random effects model to do the meta-analysis.^14^ We selected the random effects model a priori, as we expected significant between study heterogeneity. We assessed inter-study heterogeneity with the Cochran Q test and quantified it by using the I^2^ statistic. For the Q statistic, we considered a P value <0.1 to be a statistically significant indicator of heterogeneity. I^2^ values considered representative of low, moderate, and high risk of heterogeneity were <25%, 26-50%, and >50%, respectively.^15^ We assessed publication bias by using funnel plot analysis, with evaluation of asymmetry by visual inspection followed by Egger’s test.^16^

We planned a priori analyses stratified by geographical region, donor type, and population type. As estimating hazard ratios from Kaplan-Meier curves is lower down the hierarchy of evidence, we did a sensitivity analysis for the primary outcome by limiting studies to only those presenting adjusted hazard ratios with confidence intervals. Additionally, as we regarded Cox regression results reported as relative risks to be hazard ratios, assuming the authors had used inappropriate statistical terms, we did a sensitivity analysis of studies by restricting studies to only those that explicitly stated their results as hazard ratios. We did further sensitivity analyses by looking at studies on the basis of the median point of case recruitment before and after the year 2000, outlier analysis (removing studies with confidence intervals that do not overlap with the confidence intervals of the pooled effect), leave-one-out influence analysis to assess the effect of individual studies on the pooled effect,^17^ and construction of a graphical display of heterogeneity (GOSH plot). This plot is an exploratory combinatorial method that helps with visualisation of between study heterogeneity by doing all possible meta-analyses in all the different study subsets (that is, effect size of 2^n^−1 subsets).^18^ Lastly, to further investigate the heterogeneity and effect of continuous study moderators, we also planned meta-regression (using mean age, maximum duration of follow-up, and median period of case recruitment as covariates).

We used R 4.0·4 for all analyses, with packages including tidyverse, meta, and metafor.^19^ ^20^ ^21^ We defined statistical significance for a treatment effect as P<0.05, and all tests were two sided.

### Patient and public Involvement

Although this research involved no direct patient or public involvement, the research question was informed from national kidney patient group meetings in which the risk versus benefit of kidney transplantation compared with remaining on dialysis has been discussed as a key question.

## Results

### Search results

**Fig 1:**
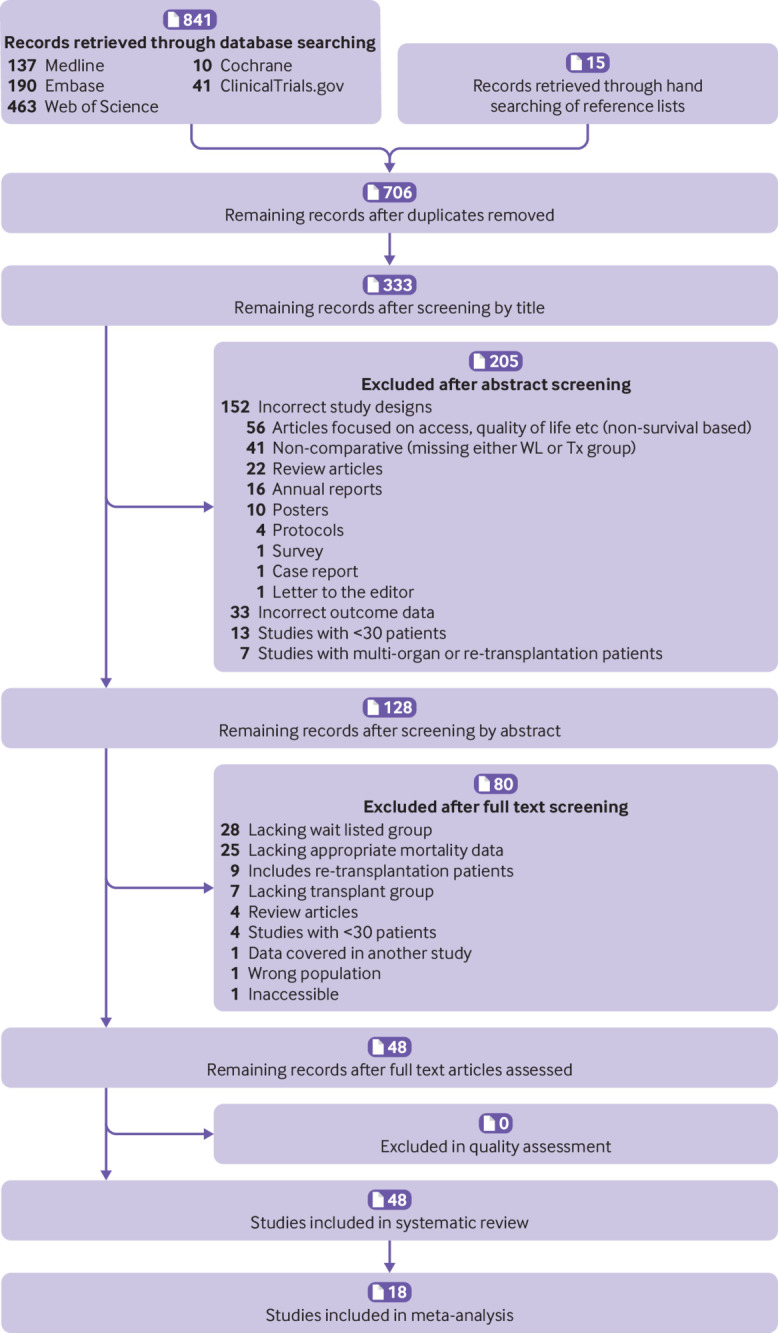
Study selection for systematic review and meta-analysis. Tx=transplantation; WL=waiting list

We identified 48 studies eligible for this systematic review.^22^ ^23^ ^24^ ^25^ ^26^ ^27^ ^28^ ^29^ ^30^ ^31^ ^32^ ^33^ ^34^ ^35^ ^36^ ^37^ ^38^ ^39^ ^40^ ^41^ ^42^ ^43^ ^44^ ^45^ ^46^ ^47^ ^48^ ^49^ ^50^ ^51^ ^52^ ^53^ ^54^ ^55^ ^56^ ^57^ ^58^ ^59^ ^60^ ^61^ ^62^ ^63^ ^64^ ^65^ ^66^ ^67^ ^68^ ^69^ After data extraction and further screening, we found 18 studies containing sufficient non-overlapping outcome data suitable for meta-analysis.^24^ ^27^ ^30^ ^31^ ^32^ ^33^ ^34^ ^36^ ^42^ ^48^ ^50^ ^52^ ^61^ ^62^ ^64^ ^66^ ^67^ ^68^ Figure 1 shows a PRISMA flow diagram detailing the process of study selection.

### Study characteristics

All 48 studies were observational cohort in design: 42 studies were retrospective (of which 37 were based on registries with prospectively collected data), five studies were prospective,^27^ ^48^ ^51^ ^52^ ^68^ and one study lacked clarification with respect to timing of data collection.^22^ Thirty eight studies were based on national or regional registry data, three were multisite studies, and seven were single site studies. Concerning the geographical representation, studies were conducted in Europe (n=24), North America (n=20), South America (n=3), and Oceania (n=1).

### Patient characteristics

Study sample sizes had large variability, ranging from 81 to 449 937 (median 2040); enrolment of study participants ranged from 1971 to 2016, and maximum duration of follow-up ranged from three to 25 years. In total, 452 119 patients underwent transplantation and 793 731 waitlisted patients remained on dialysis. In studies reporting sex, the percentage of male patients was 61% (n=144 748/235 789) in the transplantation group and 59% (n=146 991/248 394) in the waitlisted dialysis group. The mean age of patients was also comparable between the two groups at 51.1 (range 34-73) years and 51.3 (37-73) years for transplantation and dialysis patients, respectively.

### Quality of studies

The mean quality score of the eligible studies according to the NOS scale was 8.5 (range 7-9) out of 9, representing overall highly quality observational data (supplementary table B). Supplementary table C summarises the baseline study characteristics, patient characteristics, and total quality score. A detailed summary of the statistical procedures used, and covariates adjusted for, across the studies is provided in supplementary table D.

### Systematic review of evidence

In total, 92% (n=44/48) of studies reported a significant overall long term (one year or more) survival benefit from transplantation compared with dialysis. Thirty one of those 44 studies reported adjusted hazard ratios for all cause mortality (hazard ratio range 0.18-0.73; 95% confidence range 0.08 to 0.96). Eight studies reported the adjusted risk in discrete time periods,^25^ ^26^ ^30^ ^34^ ^38^ ^53^ ^59^ ^63^ and all found that mortality risk in transplant recipients was significantly higher than in waitlisted dialysis patients immediately after transplantation (hazard ratio range 1.3-17.7 at 0-30 days; 1.5-4.8 at 0-3 months). However, in all eight studies, the increased mortality risk attributable to perioperative and postoperative risk factors was eventually offset, and by one year the mortality risk was significantly lower in transplant recipients (hazard ratio range 0.19-0.49). Fourteen of the 44 studies reported adjusted relative hazards stratified by donor type. In all cases, where respective comparisons were available, studies reported living donor transplantation as conferring a greater survival benefit over deceased donor transplantation,^31^ ^35^ ^45^ ^49^ ^55^ ^59^ ^60^ ^61^ ^63^ standard criteria donors over extended criteria donors,^41^ ^47^ ^55^ ^60^ ^69^ and finally hepatitis C virus seronegative over hepatitis C virus seropositive donor transplantation.^37^ ^65^ Detailed outcome data with adjusted hazard ratios for each study and stratum are provided in supplementary table D. Benefit was found in all adults aged ≥18, older adults (≥60,^24^ ^28^ ^29^ ^38^ ^47^ ^66^ ^67^ ≥65,^28^ ^55^ ^58^ ^61^ and ≥70^45^ ^50^ ^62^), and population groups with obesity (body mass index ≥30),^35^ diabetes as a comorbidity,^43^ ^44^ ^58^ systemic sclerosis,^39^ peripheral arterial disease,^43^ ^60^ and patients seropositive for hepatitis C virus.^65^

Although 44 studies showed an overall benefit favouring transplantation, 11 of those studies identified stratums in which transplantation offered no statistically significant benefit over remaining on dialysis. No difference was reported in patients with kidney failure caused by glomerulonephritis^25^; patients aged 65-70 years^28^; patients with kidney failure caused by hypertension or a hereditary cause^30^; patients with body mass index ≥41^35^; patients aged ≥70 years with kidney failure caused by glomerulonephritis^45^; patients receiving standard criteria donor transplantation compared with those on nocturnal haemodialysis^49^; patients aged ≥70 years on dialysis between 1990 and 1999^50^; patients classified as being at low risk by the American Society of Transplantation^52^; patients with peripheral arterial disease receiving deceased donor allografts^59^; patients aged ≥70 years^67^; and patients with chronic obstructive pulmonary disease, aged ≥70 years, or with kidney failure caused by diabetes.^58^

No study described an overall lower mortality risk associated with dialysis, with 8% (n=4/48) of studies showing no difference in long term survival between treatment modalities. Two of those studies were single site studies published after 1975 but representing data from the early 1970s and likely to be non-representative of contemporary clinical practice.^22^ ^23^ One study was based across two Dutch sites representing data from only a small cohort of patients (n=156) between 1990 and 1997 with no regression analysis.^32^ Finally, one study presented data on patients aged ≥70 years from a French registry between 2002 and 2013.^63^ Although this study found that risk in the transplantation group had halved by nine months compared with the dialysis group, it was not enough to offset the high perioperative risk associated with transplantation by the end of the study period (month 36).

### Meta-analysis

**Fig 2:**
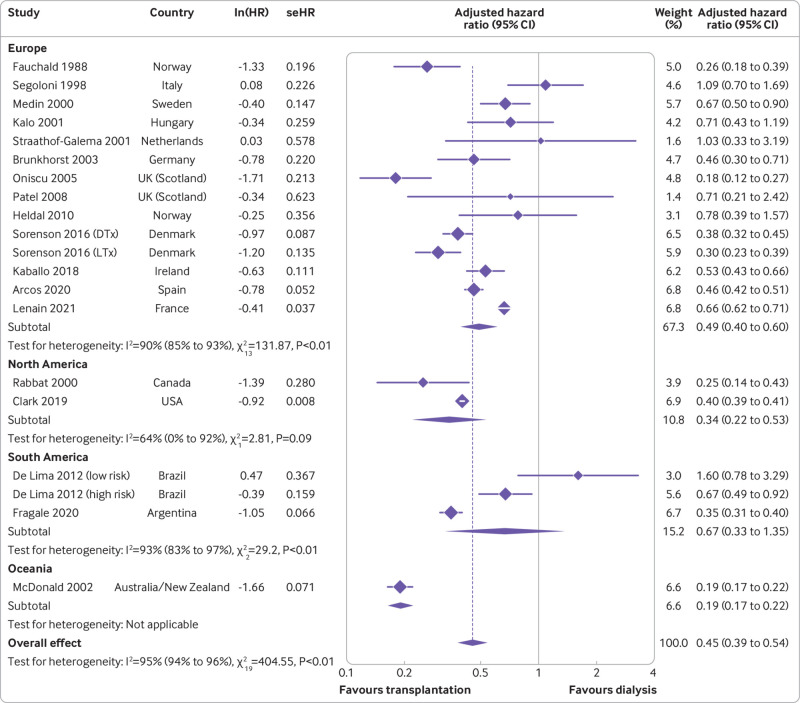
Forest plot of hazard ratios (with 95% confidence interval (CIs)) for transplantation versus waitlisted dialysis, stratified by geographical region. lnHR=log hazard ratio; seHR=standard error hazard ratio

Of the 18 studies included in the meta-analysis, 11 studies presented adjusted regression models reporting both risk (hazard ratio or relative risk) and precision (standard error, P value, or confidence intervals).^30^ ^31^ ^34^ ^36^ ^42^ ^50^ ^61^ ^62^ ^64^ ^66^ ^67^ Hazard ratios were not directly reported in seven studies and were extrapolated from the reported Kaplan-Meier survival curves. Overall, the pooled estimate showed that transplantation was associated with a 55% lower long term mortality risk in patients with kidney failure compared with dialysis (hazard ratio 0.45, 95% confidence interval 0.39 to 0.54; P<0.001), as shown in fig 2. Heterogeneity was significant by the Q statistic (405, df=19; P<0.01) and by I^2^ (95.3%; P<0.01).

### Subgroup analyses

**Fig 3:**
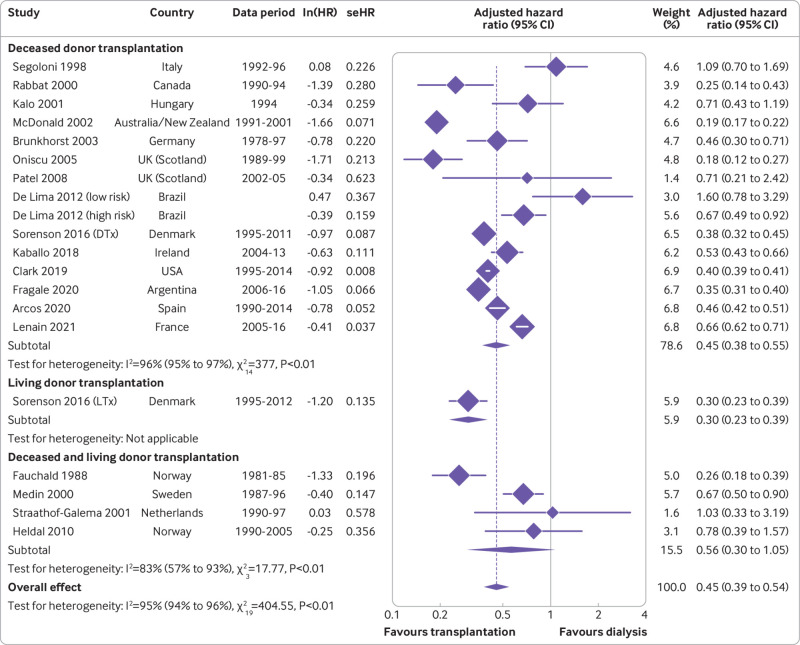
Forest plot of hazard ratios (with 95% confidence interval (CIs)) for transplantation versus waitlisted dialysis, stratified by donor type. lnHR=log hazard ratio; seHR=standard error hazard ratio

**Fig 4:**
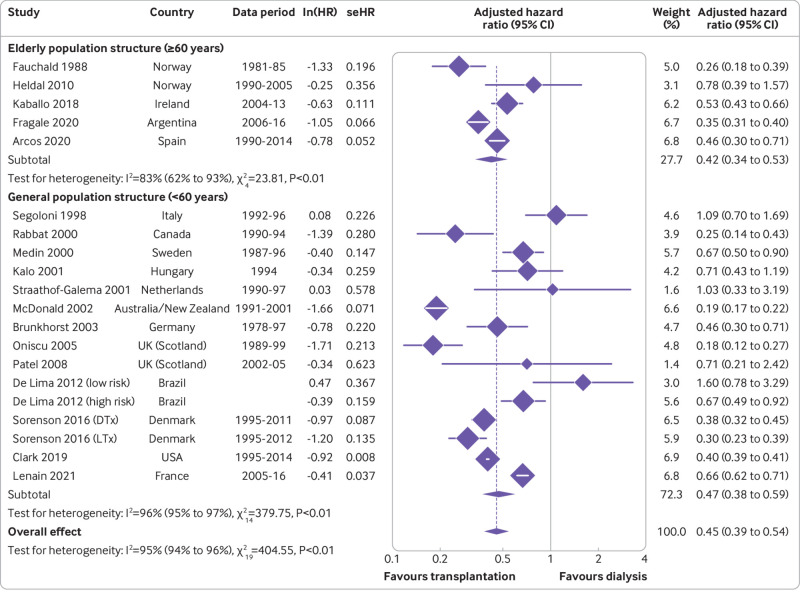
Forest plot of hazard ratios (with 95% confidence interval (CIs)) for transplantation versus waitlisted dialysis, stratified by population type. lnHR=log hazard ratio; seHR=standard error hazard ratio

To investigate the significant heterogeneity, we stratified studies by geographical region (continent; fig 2), donor type (living versus deceased; fig 3), and population type (general versus aged ≥60 years; fig 4). The pooled hazard ratio for long term, all cause mortality for the transplantation group, compared with the dialysis group, was lowest in Oceania (n=1; hazard ratio 0.19, 0.17 to 0.22), followed by North America (n=2; 0.34, 0.22 to 0.53; Cochran Q statistic 2.81, P=0.09; I^2^=64.4%, P<0.01) and Europe (n=13; 0.49, 0.40 to 0.60; Cochran Q statistic 131.87, P<0.01; I^2^=90.1%, P<0.01), which were comparable. However, the result between the two modalities was not statistically different in South America (n=2; 0.67, 0.33 to 1.35; Cochran Q statistic=29.2, P<0.01; I^2^=93.2%, P<0.01). Although subgroup analyses by continent helped to explain some of the heterogeneity, it remained largely significant. Stratification by donor type showed living donor transplantation to have a lower long term mortality risk compared with deceased donor transplantation, with hazard ratios of 0.30 (0.23 to 0.39) and 0.45 (0.38 to 0.55), respectively. However, the result was not significant owing to overlapping confidence intervals and only a single study contributing data for living donor transplantation. Furthermore, this analysis was also unable to account for the significant heterogeneity present. Finally, stratification by population type showed a similar trend to the previous subgroup analyses, with both the general (hazard ratio 0.47, 0.38 to 0.59) and ≥60 year group (0.42, 0.34 to 0.53) showing transplantation as conferring a lower mortality risk compared with dialysis; however, stratification failed to elucidate the cause of the significant heterogeneity.

### Sensitivity analyses

Sensitivity analyses that restricted contributing studies to only those presenting adjusted hazard ratios (n=11) and removing those for which we derived hazard ratios graphically showed similar findings (hazard ratio 0.37, 0.31 to 0.44; Cochran Q statistic 163.65, P<0.01; I^2^=93.3%, P<0.01). Further restricting the 11 studies to only those in which we did not assume that the provided Cox regression model results were hazard ratios when presented as relative risks (that is, the hazard ratios were explicitly clear; n=6) made no significant change to the result (hazard ratio 0.35, 0.29 to 0.43; Cochran Q statistic 127.26, P<0.01; I^2^=95.3%, P<0.01).

To evaluate period effects, we calculated a pooled hazard ratio for studies with the median point of case recruitment on or before the year 2000 (n=10; hazard ratio 0.44, 0.28 to 0.71) and after the year 2000 (n=7; 0.44, 0.36 to 0.53), which showed no significant period effect.

**Fig 5:**
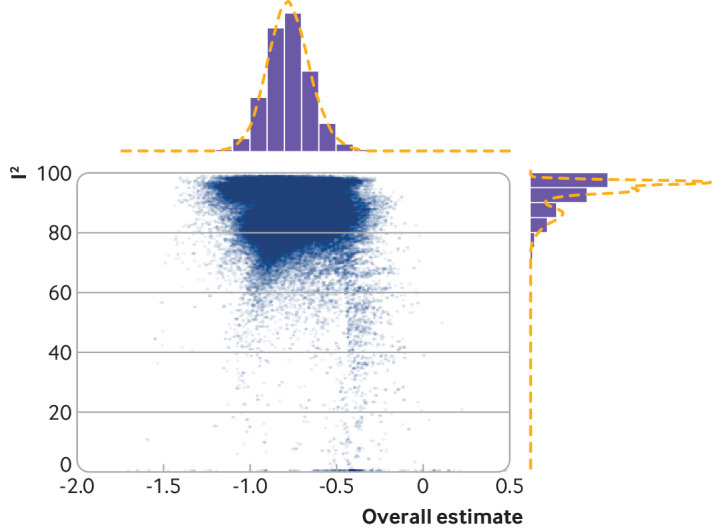
GOSH plot (graphical display of heterogeneity) of I^2^ against summary effect sizes (log hazard ratio) indicating unimodal effect measure but bimodal heterogeneity pattern. Regardless of study selection, heterogeneity remains significant

For the outlier analysis, we identified five studies as outliers and consequently removed them. This had no effect on the pooled estimate (hazard ratio 0.44, 0.39 to 0.49) and a marginal effect on heterogeneity, which remained significant (Cochran Q statistic 65.31, P<0.001; I^2^=78.6%). Similarly, leave-one-out analysis showed that no singular study when omitted altered the significance of the pooled hazard ratio or diminished the significant heterogeneity present (supplementary table E). Finally, we constructed a GOSH plot. For overall effect size, we saw a unimodal distribution suggesting homogeneity in the pooled estimate, with transplantation offering lower mortality risk compared with dialysis regardless of the subset of studies selected. However, we saw a bimodal distribution for I^2^ results suggesting the presence of a more homogenous cluster of studies. Nevertheless, even with the most optimal subset of studies selected for meta-analysis, heterogeneity was still significant at I^2^≥70 (fig 5).

### Meta-regression

We did a meta-regression analysis to estimate the effect of continuous study moderators such as mean age, maximum duration of follow-up, and median period of data collection. None of these could account for the overall heterogeneity (supplementary table F).

### Publication bias

The funnel plot was broadly symmetrical, and this was confirmed by a non-significant Egger’s test (0.8821, P=0.46), suggesting a low probability of publication bias (supplementary figure A).

## Discussion

This systematic review compared all cause mortality associated with transplantation versus dialysis in waitlisted patients with kidney failure, with overwhelming evidence showing transplantation to be associated with significant survival benefit compared with dialysis. However, although the overall benefit of transplantation for patients with kidney failure is a homogenous message, analysis for various subgroups was conflicting, failing to show a clear survival benefit of transplantation versus dialysis for selected demographics. Our review also identified significant heterogeneity in the published literature, which was retained despite further statistical manipulation, suggesting that population level data should be cautiously translated to individual patients for personalised decision making.

### Implications of findings

The sources of heterogeneity in our study are potentially wide ranging including, but not limited to, geographical location, statistical techniques, donor type, dialysis modality, comorbidity burden, time coverage, immunosuppression, healthcare infrastructure, and basic demographical variables. We attempted to account for some of these factors, but none showed significance. Poor reporting and stratification of these variables within the available studies limited deeper analyses, but management of patients with kidney failure being a complex multivariate equation extending beyond the covariates measured is more likely.

Appreciating that the risk-benefit ratio of transplantation versus dialysis is not just a determination about survival advantage is important. The impact on quality of life is also an important consideration in assessment of candidates for kidney transplantation.^70^ Our work provides important data at a population level to inform counselling about survival, but personalising risk-benefit decisions at an individual level remains a clinical challenge. Although regulatory guidance has always encouraged involvement of patients in decision making, the Montgomery ruling has altered the legal basis of obtaining informed consent in countries such as the UK.^71^ The effect of individual clinical circumstances must now be factored into the probability of benefit or harm occurring. Therefore, although we believe that our systematic review of population cohort studies is informative for risk counselling of candidates for kidney transplantation, it simply provides supportive information to an overall determination of personalised risk for individuals.

### Strengths and limitations of study

This is the most up-to-date systematic review concerning mortality in transplantation versus dialysis and the first to focus on primary kidney transplantation versus waitlisted candidates, thereby reducing the selection bias present in previous systematic analyses. Our review adhered to the full systematic review protocol, with no limitation on language and dual screening of titles, abstracts, full text, and risk of bias assessment. Consequently, we were able to identify five additional studies that were missed by the previous systematic review by Tonelli and colleagues and 20 new additional studies published after it. Furthermore, this study is the first to present a meta-analysis of time-to-event data in accordance with the Cochrane Handbook for Systematic Reviews of Interventions.

The limitations of this work are worth noting. Firstly, substantial heterogeneity existed in the published literature, which could not be accounted for despite appropriate analytical techniques. Secondly, in the absence of studies reporting adjusted hazard ratios with precision estimates, we included studies that reported Kaplan-Meier curves using the palmar technique by extrapolating hazard ratios based on the minimum and maximum follow-up periods and the censoring pattern. However, digitalisation and extrapolation of hazard ratios from Kaplan-Meier curves can be problematic in terms of reproducibility and inaccuracies. Nevertheless, sensitivity analyses with exclusion of such studies found no significant systematic biases. Thirdly, we included studies ranging over many decades (1977-2021), during which significant advancements in transplantation and dialysis have been made. However, sensitivity analysis for earlier and more recent studies (>2000 and ≤2000) showed that point estimates were similar, and meta-regression showed no significant effect of era either. Fourthly, we considered long term mortality as risk after one year; this encapsulates many different time interval periods and limits data interpretation. However, on GOSH plot construction, a unimodal distribution was seen for effect size, showing consistency across the studies regardless of which subset was selected. The large I^2^ value (95.3%) indicates a wide range of plausible risk estimates, but we found no evidence in the systematic review of dialysis offering greater longer term survival than transplantation—at best, risk equivalence was reported. Finally, our study exclusively explored mortality benefit, and any other clinical patient reported outcome such as quality of life or health economic difference was beyond the scope of this work.

### Conclusions

To conclude, evidence from our systematic review overwhelmingly found that transplantation was associated with significant reduction in long term mortality risk compared with remaining on dialysis for waitlisted patients with kidney failure. Despite a uniform estimate of mortality risk, significant heterogeneity existed between studies that limits external validity, especially for selected cohorts such as older candidates or personalised decision making. Therefore, although we can confidently conclude that transplantation is the modality of choice for patients with kidney failure, determining subgroups of patients in which transplantation facilitates the greatest utility remains difficult and needs further investigation.

## What is already known on this topic

For people living with or approaching kidney failure, kidney transplantation provides superior survival to dialysis therapy for most patients
Most patients with advanced chronic kidney disease or living on dialysis are not active on kidney transplant waiting lists
With evolving clinical practice, important questions remain about the survival benefit for patients with kidney failure in the contemporary era

## What this study adds

The overall survival benefit observed with kidney transplantation in the systematic review is a homogenous message
However, analysis by various subgroups is conflicting, failing to show a clear survival benefit of transplantation versus dialysis for selected demographics
Targeted counselling is warranted to ensure that every patient with kidney failure is given a fair and equitable opportunity to be assessed as a candidate for kidney transplantation

## Data Availability

Extracted data are available from the corresponding author on request.

## Web extra

Extra material supplied by authors

Web appendix: Supplementary materials

## Notes

Contributors: DC and AS designed the study. DC, JP, and AS planned the statistical analysis. DC and AC analysed and extracted data, with assistance from JP. DC and AC assessed study eligibility and quality. JP and AS monitored the review process and shared responsibility for coding direct and indirect interventions. DC and AS wrote the first draft of the manuscript. All authors contributed to the interpretation and subsequent edits of the manuscript. AS is the guarantor. The corresponding author attests that all listed authors meet authorship criteria and that no others meeting the criteria have been omitted.

Dissemination to participants and related patient and public communities: Strategies to disseminate the research findings include abstract presentations at scientific congresses to nephrology and transplantation professionals. Patient advocacy groups and kidney charities will be informed with a plain language summary of the study findings. Social media platforms (for example, @da_chaudhry, @AdnanSharif1979) will be used to disseminate study findings to a wider audience and media engagement to support any press releases.

Provenance and peer review: Not commissioned, externally peer reviewed.
