# Supplementary material for: Survival for waitlisted kidney failure patients receiving transplantation versus remaining on waiting list: systematic review and meta-analysis
Source: BMJ. 2022 Mar 1;376:e068769. doi: 10.1136/bmj-2021-068769 (PMC8886447; doi:10.1136/bmj-2021-068769)
Supplement: Supplementary file 1 — Web appendix: Supplementary materials [file chad068769.ww1.pdf]

Supplementary Table A. Detailed Search Methodology

Search methods

The databases Medline (via Ovid), Embase (via Ovid), Cochrane Library (including the Central Registry of Controlled Trials), and Web of Science (Core Collection) were searched from inception to March 2021 (01/03/2021). They were searched using a mixture of free text terms, and thesaurus terms (when available). Search syntax was adapted to code for the individual database platforms. See the Appendix for the exact search terms used in each database.

Results

Database	Results
Medline via Ovid	137
Embase via Ovid	190
Cochrane (including Central)	51 (of which 41 were trials)
Web of Science (Core Collection)	463

After deduplication: 699 unique results

Appendix: exact search terms used in each database

Medline via Ovid

1. Chronic kidney disease.ti,ab,kw.
2. Chronic kidney failure.tw,ab,kw.
3. Chronic Kidney Insufficien$.tw,ab,kw.
4. Chronic Nephropathy.tw,ab,kw.
5. Chronic Renal Disease.tw,ab,kw.
6. Chronic renal failure.tw,ab,kw.
7. CRF.tw,ab,kw.
8. Chronic Renal Insufficien$.tw,ab,kw.
9. End-stage renal disease.tw,ab,kw.
10. End-stage renal failure.tw,ab,kw.
11. End-stage kidney disease.tw,ab,kw.
12. End-stage kidney failure.tw,ab,kw.
13. ESRD.tw,ab,kw.
14. ESRF.tw,ab,kw.
15. Kidney Chronic Failure.tw,ab,kw.
16. Kidney Failure, Chronic/
17. Kidney Insufficien$.tw,ab,kw.
18. Kidney failure.tw,ab,kw. or Renal Insufficiency
19. Renal Insufficiency, Chronic/
20. Terminal Kidney Failure.tw,ab,kw.
21. Kidney$ Allograft$.tw,ab,kw.
22. Kidney Transplantation/
23. ((kidney$ or renal) adj5 (transplant$ or graft$ or donor$ or recipient$ or replac$ or artificial$ or extracorpeal$)).tw,ab,kw.
24. Hemofiltration/
25. Hemofiltrat$.tw,ab,kw.
26. Kidneys, Artificial/
27. kidney dialy$.tw,ab,kw.

28. Kidney$ replacement therap$.tw,ab,kw.
29. Peritoneal Dialysis/
30. predialy$.tw,ab,kw.
31. pre-dialy$.tw,ab,kw.
32. exp Renal Dialysis/
33. Renal Replacement Therapy/
34. Ultrafiltration/
35. ultrafiltrat$.tw,ab,kw.
36. wait-listed.tw,ab,kw.
37. wait listed.tw,ab,kw.
38. waiting listed.tw,ab,kw.
39. patient listed.tw,ab,kw.
40. Waiting Lists/
41. Transplant list.tw,ab,kw.
42. death.tw,ab,kw.
43. mortality.tw,ab,kw.
44. exp Mortality/
45. "survival analysis".tw,ab,kw.
46. Survival Analysis/
47. Survival Rate/
48. 1 or 2 or 3 or 4 or 5 or 6 or 7 or 8 or 9 or 10 or 11 or 12 or 13 or 14 or 15 or 16 or 17 or 18 or 19 or 20
49. 21 or 22 or 23
50. 24 or 25 or 26 or 27 or 28 or 29 or 30 or 31 or 32 or 33 or 34 or 35
51. 36 or 37 or 38 or 39 or 40 or 41
52. 42 or 43 or 44 or 45 or 46 or 47
53. 48 and 49 and 50 and 51 and 52

Embase via Ovid

1. chronic kidney disease.tw,ab,kw.
2. Chronic kidney failure.tw,ab,kw.
3. chronic kidney failure/
4. Chronic Kidney Insufficien$.tw,ab,kw.
5. Chronic Nephropathy.tw,ab,kw.
6. Chronic Renal Disease.tw,ab,kw.
7. Chronic renal failure.tw,ab,kw.
8. CRF.tw,ab,kw.
9. Chronic Renal Insufficien$.tw,ab,kw.
10. End-stage renal disease.tw,ab,kw.
11. End-stage renal failure.tw,ab,kw.
12. End-stage kidney disease.tw,ab,kw.
13. End-stage kidney failure.tw,ab,kw.
14. ESRD.tw,ab,kw.
15. ESRF.tw,ab,kw.
16. Kidney Chronic Failure.tw,ab,kw.
17. chronic kidney failure/
18. Kidney Insufficien$.tw,ab,kw.
19. Renal insufficiency.tw,ab,kw.
20. Terminal Kidney Failure.tw,ab,kw.
21. kidney failure/
22. kidney allograft/

23. kidney transplantation/
24. ((kidney$ or renal) adj5 (transplant$ or graft$ or donor$ or recipient$ or replac$ or artificial$ or extracorpeal$)).tw,ab,kw.
25. hemofiltration/
26. Hemofiltrat$.tw,ab,kw.
27. artificial kidney.tw,ab,kw.
28. kidney dialy$.tw,ab,kw.
29. Kidney$ replacement therap$.tw,ab,kw.
30. peritoneal dialysis/
31. predialy$.tw,ab,kw.
32. pre-dialy$.tw,ab,kw.
33. hemodialysis/
34. Renal dialysis.tw,ab,kw.
35. renal replacement therapy/
36. ultrafiltration/
37. ultrafiltrat$.tw,ab,kw.
38. wait-listed.tw,ab,kw.
39. wait listed.tw,ab,kw.
40. waiting listed.tw,ab,kw.
41. patient listed.tw,ab,kw.
42. waiting list.tw,ab,kw.
43. Transplant list.tw,ab,kw.
44. death/
45. death.tw,ab,kw.
46. mortality/
47. mortality.tw,ab,kw.
48. "survival analysis".tw,ab,kw.
49. survival analysis/
50. survival rate/
51. 1 or 2 or 3 or 4 or 5 or 6 or 7 or 8 or 9 or 10 or 11 or 12 or 13 or 14 or 15 or 16 or 17 or 18 or 19 or 20 or 21
52. 22 or 23 or 24
53. 25 or 26 or 27 or 28 or 29 or 30 or 31 or 32 or 33 or 34 or 35 or 36 or 37
54. 38 or 39 or 40 or 41 or 42 or 43
55. 44 or 45 or 46 or 47 or 48 or 49 or 50
56. 51 and 52 and 53 and 54 and 55

Cochrane Library (including the Cochrane Central Register of Controlled Trials)

1. (Chronic kidney disease OR Chronic kidney failure OR Chronic Kidney Insufficien* OR Chronic Nephropathy OR Chronic Renal Disease OR Chronic renal failure OR CRF OR Chronic Renal Insufficien* OR End-stage renal disease OR End-stage renal failure OR End-stage kidney disease OR End-stage kidney failure OR ESRD OR ESRF OR Kidney Chronic Failure OR Kidney Insufficien* OR Kidney failure OR Terminal Kidney Failure):ti,ab,kw (Word variations have been searched)
2. MeSH descriptor: [Kidney Failure, Chronic] this term only
3. MeSH descriptor: [Renal Insufficiency, Chronic] this term only
4. (Kidney Allograft OR kidney transplantation OR Kidney transplant OR Renal transplant OR Kidney graft OR Renal graft OR Kidney donor OR Renal donor OR Kidney recipient OR Renal recipient):ti,ab,kw (Word variations have been searched)

5. MeSH descriptor: [Kidney Transplantation] this term only
6. (Hemofiltrat* OR hemofiltration OR dialysis OR Kidney dialy* OR Peritoneal dialysis OR predialy* OR pre-dialy* OR Renal replacement therapy OR Ultrafiltration OR ultrafiltrat* OR Artificial kidney):ti,ab,kw (Word variations have been searched)
7. MeSH descriptor: [Renal Dialysis] explode all trees
8. MeSH descriptor: [Renal Replacement Therapy] this term only
9. (wait-listed OR wait listed OR waiting listed OR patient listed Or Waiting list OR Transplant list):ti,ab,kw (Word variations have been searched)
10. MeSH descriptor: [Waiting Lists] this term only
11. (death OR mortality OR survival analysis OR "survival analysis" OR survival rate):ti,ab,kw (Word variations have been searched)
12. MeSH descriptor: [Mortality] this term only
13. MeSH descriptor: [Survival Rate] this term only
14. MeSH descriptor: [Survival Analysis] explode all trees
15. #1 OR #2 OR #3
16. #4 OR #5
17. #6 OR #7 OR #8
18. #9 OR #10
19. #11 OR #12 OR #13 OR #14
20. #15 AND #16 AND #17 AND #18 AND #19

Web of Science (Core Collection)

1. (TS=(Chronic kidney disease OR Chronic kidney failure OR Chronic Kidney Insufficien* OR Chronic Nephropathy OR Chronic Renal Disease OR Chronic renal failure OR CRF OR Chronic Renal Insufficien* OR End-stage renal disease OR End-stage renal failure OR End-stage kidney disease OR End-stage kidney failure OR ESRD OR ESRF OR Kidney Chronic Failure OR Kidney Insufficien* OR Kidney failure OR Terminal Kidney Failure)) AND DOCUMENT TYPES: (Article)
2. (TS=(Kidney Allograft OR kidney transplantation OR Kidney transplant OR Renal transplant OR Kidney graft OR Renal graft OR Kidney donor OR Renal donor OR Kidney recipient OR Renal recipient)) AND DOCUMENT TYPES: (Article)
3. (TS=(Hemofiltrat* OR hemofiltration OR dialysis OR Kidney dialy* OR Renal dialysis OR Peritoneal dialysis OR predialy* OR pre-dialy* OR Renal replacement therapy OR Ultrafiltration OR ultrafiltrat* OR Artificial kidney)) AND DOCUMENT TYPES: (Article)
4. (TS=(wait-listed OR wait listed OR waiting listed OR patient listed Or Waiting list OR Transplant list)) AND DOCUMENT TYPES: (Article)
5. (TS=(death OR mortality OR survival analysis OR "survival analysis" OR survival rate)) AND DOCUMENT TYPES: (Article)
6. (#5 AND #4 AND #3 AND #2 AND #1) AND DOCUMENT TYPES: (Article)

<u>Supplementary Table B.</u> Newcastle-Ottawa Scale scores for all studies.

Authors	Year	Representativeness of the exposed cohort	Selection of the non-exposed cohort	Ascertainment of exposure	Demonstration that outcome of interest not present at start	Comparability: age and sex	Comparability: other factors (Wait listed / Co-morbidities etc.)	Assessment of outcome	Follow-up long enough (≥ 1 year)	Adequacy of follow-up	Total NOS score (/9)
Cantaluppi et al.	1977	1	1	1	1	1	0	1	1	1	8
Golper et al.	1978	1	1	1	1	0	0	1	1	1	7
Fauchald et al.	1988	1	1	1	1	0	0	1	1	1	7
Port et al.	1993	1	1	1	1	1	1	1	1	1	9
Ojo et al.	1994	1	1	1	1	1	1	1	1	1	9
Bonal et al.	1997	1	1	1	1	1	1	1	1	1	9
Segoloni et al.	1998	1	1	0	1	1	1	0	1	1	7
Wolfe et al.	1999	1	1	1	1	1	1	1	1	1	9
Medin et al.	2000	1	1	1	1	1	1	1	1	1	9
Rabbat et al.	2000	1	1	1	1	0	0	1	1	1	7
Kalo et al.	2001	1	1	1	1	1	0	1	1	1	8
Straathof-Galema et al.	2001	1	1	1	1	1	1	1	1	1	9
McDonald et al.	2002	1	1	1	1	1	1	1	1	1	9
Brunkhorst et al.	2003	1	1	1	1	1	1	1	1	1	9
Glanton et al.	2003	1	1	1	1	1	1	1	1	1	9
Abbott et al.	2004	1	1	1	1	1	1	1	1	1	9
Gibney et al.	2004	1	1	1	1	1	1	1	1	1	9
Oniscu et al.	2004	1	1	1	1	0	0	1	1	1	7
Gill et al.	2005	1	1	1	1	1	1	1	1	1	9
Merion et al.	2005	1	1	1	1	1	1	1	1	1	9
Oniscu et al.	2005	1	1	1	1	1	1	1	1	1	9
Snyder et al.	2006	1	1	1	1	1	1	1	1	1	9

Gill et al.	2007	1	1	1	1	1	1	1	1	1	9
Rao et al.	2007	1	1	1	1	1	1	1	1	1	9
Savoye et al.	2007	1	1	1	1	1	1	1	1	1	9
Sorensen et al.	2007	1	1	1	1	0	0	1	1	1	7
Patel et al.	2008	1	1	1	1	0	0	1	1	1	7
Navarro et al.	2009	1	1	1	1	1	1	0	1	1	8
Pauly et al.	2009	1	1	1	1	1	1	1	1	1	9
Heldal et al.	2010	1	1	1	1	1	1	1	1	1	9
Kumar et al.	2011	1	1	1	1	0	0	1	1	1	7
Bisigniano et al.	2012	1	1	1	1	1	1	1	1	1	9
De Lima et al.	2012	1	1	1	1	0	0	1	1	1	7
Bouaoun et al.	2013	1	1	1	1	1	1	1	1	1	9
Gill et al.	2013	1	1	1	1	1	0	1	1	1	8
Schold et al.	2014	1	1	1	1	1	1	1	1	1	9
Lloveras et al.	2015	1	1	1	1	1	1	1	1	1	9
Brar et al.	2016	1	1	1	1	1	1	1	1	1	9
Cassuto et al.	2016	1	1	1	1	1	1	1	1	1	9
Perez-Saez et al.	2016	1	1	1	1	1	1	1	1	1	9
Sorensen et al.	2016	1	1	1	1	1	1	1	1	1	9
Kaballo et al.	2018	1	1	1	1	1	1	1	1	1	9
Legeai et al.	2018	1	1	1	1	1	1	1	1	1	9
Clark et al.	2019	1	1	1	1	0	0	1	1	1	7
Sawinski et al.	2019	1	1	1	1	0	0	1	1	1	7
Arcos et al.	2020	1	1	1	1	1	1	1	1	1	9
Fragale et al.	2020	1	1	1	1	1	1	1	1	1	9
Lenain et al.	2021	1	1	1	1	1	1	1	1	1	9
Hellemans et al.	2021	1	1	1	1	1	1	1	1	1	9

Supplementary Table C. Baseline characteristics and overall outcomes of included studies.

Study Characteristics						*Population type*								*Outcomes and quality*	
Author and year	***Country***	***Study design***	***Data source***	***Inclusion period***	***Follow-up (years)***	***Population type***	***Waitlist group sample size***	***Transplant group sample size***	***Donor type***	***Dialysis type***	***Mean Age ± SD in years (range) – WL***	***Mean Age ± SD in years (range) – Tx***	***Male (%)***	***For Long-Term Survival Transplantation better than dialysis (Y/N)***	***NOS score***
Cantaluppi et al. (1977)	Italy	Cohort study	Single site	≥1972	Max: 5	–	61	66	DTx and LTx (grouped)	Home HD	37 ± 10 (19-52)	34 ± 9 (17-54)	66% (WL) 71% (Tx)	N	8
Golper et al. (1978)	US	Cohort Study	Single site	1971-1977	Max: 6.8	≥45 year	51	30	DTx	NR	51 ± 4	51 ± 3	NR	N	7
Fauchald et al. (1988)	Norway	Cohort Study	Multiple sites	1981-1985	Max: 6	≥60 years	127	122 (96 DTx, 26 LTx)	DTx and LTx	HD and CAPD	65.9	65.9	NR	Y	7
Port et al. (1993)	US	Cohort study (registry)	Michigan Kidney Registry	1984-1989	Max: 6	<65 years	770	799	DTx	NR	NR	NR	60% (Overall)	Y<br>N (KF-GN)	9
Ojo et al. (1994)	US	Cohort study (registry)	Michigan Kidney Registry	1984-1989	Max: 6	<65 years	534	236	DTx	NR	Median: 39.9	Median: 40.2	60.6% (WL) 66.1% (Tx)	Y	9
Bonal et al. (1997)	Spain	Cohort Study (registry)	RMRC	1984-1993	Max: 10	55-70 years	395	157	DTx	HD only	60.8	61.6	61.8% (WL) 57.8% (Tx)	Y (50–59-years)<br>N (65-70 years)	9
Segoloni et al. (1998)	Italy	Cohort study	Single site	1992-1996	Max: 5	–	916	344	NR	NR	46	45.4	64.3% (WL) 59.9% (Tx)	Y (not statistically significant)	7
Wolfe et al. (1999)	US	Cohort study (registry)	USRDS	1991-1996	Max: 7	<70 years	22,889	23,275	DTx	NR	Median: 40-59 years	Median: 40-59 years	60.5% (Overall) 63% (Tx) 58% (Dx)	Y	9
Medin et al. (2000)	Sweden	Cohort study	Single site	1987-1996	Max: 10	–	170	426 (DTx)	DTx and LTx	HD and PD	49 ± 13 (DTx and WL)	47 ± 13 (DTx)	NR	Y (LTx > DTx)	9
								197 (LTx)				40 ± 13	NR		
Rabbat et al. (2000)	Canada	Cohort study (registry)	CORR and MORE	1990-1994	Max: 6	–	1156	722	DTx	NR	44.3	41.9	62.8% (WL) 63.4% (Tx)	Y<br>N (KF-HTN)<br>N (KF-Hereditary)	7
Kalo et al. (2001)	Hungary	Cohort study (registry)	Hungarian subset of the ERA-EDTA	1994	Max: 3	–	430	242	DTx	HD	41.9 ± 12.3	49.0 ± 11.7	60.9% (WL) 54.1% (Tx)	Y	8
Straathof-Galema et al. (2001)	Netherlands	Cohort study	Two sites	1990-1997	Max: 7	–	54	102	DTx and LTx	HD	48.1	49.8	61.8% (WL) 61.1% (Tx)	N	9
McDonald et al. (2002)	Australia; New Zealand	Cohort study (registry)	ANZDATA	1991-2001	Max: 10	>15 - <65 years	2782	2362	DTx	HD and PD	46.2	43.8	58% (WL) 63% (Tx)	Y	9
Brunkhorst et al. (2003)	Germany	Cohort Study (registry)	Regional registry	1978-1997	Max: 19	KF-DM1	46	46	DTx	HD only	43 ± 4.96	45 ± 10.07	67.4% (WL) 67.4% (Tx)	Y	9
Glanton et al. (2003)	US	Cohort study (registry)	USRDS	1995-1999	Max: 7	Obese (BMI ≥ 30 km/m2)	5172	1719 DTx 552 LTx	DTx and LTx	HD and PD	NR	NR	54.4% (Overall)	Y (BMI = 30 kg/m2; LTx > DTx)<br>N (BMI ≥41 kg/m2)	

Study Characteristics						Population type								Outcomes and quality	
Author and year	*Country*	*Study design*	*Data source*	*Inclusion period*	*Follow-up (years)*	*Population type*	*Waitlist group sample size*	*Transplant group sample size*	*Donor type*	*Dialysis type*	*Mean Age ± SD in years (range) – WL*	*Mean Age ± SD in years (range) – Tx*	*Male (%)*	*For Long-Term Survival Transplantation better than dialysis (Y/N)*	*NOS score*
						nObese	16,896	4795 DTx 1528 LTx					NR	Y	
Abbott et al. (2004)	US	Cohort study (registry)	USRDS and UNOS	1995-2000	Max: 6	HCV+ Donor	17,094	389	DTx (HCV+ Donor)	HD and PD	50.1 ± 12.7	51.2 ± 11.3	59.9% (WL) 75.3% (Tx HCV+ Donor)	Y (HCV- > HCV+)	9
						HCV- Donor	“”	16,595					62.9% (Tx HCV- Donor)	Y	
Gibney et al. (2004)	US	Cohort study (registry)	UNOS	1985-2002	Max: 6.8	Systemic sclerosis patients	116	115	DTx and LTx	HD	–	52 ± 10	22% (Overall)	Y	9
Oniscu et al. (2004)	UK (Scotland)	Cohort study (registry)	Scottish renal registry and UK transplant databases	1989-1999	Max: 11	≥60 years	197	128	NR	HD and PD	Median: 66.3 (IQR: 63.0-72.9)	Median: 64.0 (IQR: 58.5-69.5)	59.4 (WL) 84.8 (Tx)	Y	7
Gill et al. (2005)	US	Cohort Study (registry)	USRDS	1995-2000	Max: 6	< 75 years	35,549	19,666	DTx	NR	49 ± 13	46 ± 14	58% (WL) 62% (Tx)	Y	9
Merion et al. (2005)	US	Cohort study (registry)	SRTR	1995-2002	Max: 10	ECD	45,082	7790	SCD, ECD and LTx	NR	40-59	40-59	58.1% (WL) 62.2 (ECD)	Y	9
						SCD	“”	41,052				Median: 40-59	60.6 (SCD)	Y (SCD > ECD)	
Oniscu et al. (2005)	UK (Scotland)	Cohort study (registry)	Scottish renal registry and UK transplant databases and case-note reviews	1989-1999	Max: 11	–	641	1095	DTx	HD and PD	52.77 ± 12.92	42.98 ± 13.56	61.8 (WL) 61.2 (Tx)	Y	9
Snyder et al. (2006)	US	Cohort Study (registry)	USRDS	1995-2003	–	≥ 18 years DM	19,107	11,418 (3% pre-emptive)	DTx	NR	Median: 50-64	Median: 50-64	59% (WL) 60% (Tx)	Y– Patients with PAD Y – Patients without PAD	9
						≥ 18 years nDM	34,202	32,009 (3% pre-emptive)			Median: 35-49	Median: 35-49	60% (Tx)	Y– Patients with PAD Y – Patients without PAD	
Gill et al. (2007)	US	Cohort study (registry)	USRDS	1995-2003	NR	>18 years	41,769	47,433	DTx and LTx (grouped)	HD and PD	–	49.6 ± 13.2	60.1% (Overall) 61.7% (Tx) 58.3% (Dx)	Y	9
Rao et al. (2007)	US	Cohort study (registry)	SRTR	1990-2004	Max: 15	≥70 years	3229	1390 DTx 688 ECD	DTx and ECD	NR	Median: 70-74	Median: 70-74	66.5% (WL) 70.1% (Tx)	Y (LTx > ECD) N (KF-GN)	9
Savoye et al. (2007)	France	Cohort study (registry)	National registry (REIN from 2002 onwards)	1996-2004	Mean: 2.9 ± 2.4	≥60 years	746	1962	SCD and ECD	NR	NR	NR	64% (Overall)	Y (SCD > ECD)	9
Sorensen et al.	Denmark	Cohort study	DNR and the	1994-2005	Max: 12	DM	211	114	DTx and	HD and	48.5 ± 12	40.8 ± 14	NR	Y	7

Study Characteristics						Population type								Outcomes and quality	
Author and year	*Country*	*Study design*	*Data source*	*Inclusion period*	*Follow-up (years)*	*Population type*	*Waitlist group sample size*	*Transplant group sample size*	*Donor type*	*Dialysis type*	*Mean Age ± SD in years (range) – WL*	*Mean Age ± SD in years (range) – Tx*	*Male (%)*	*For Long-Term Survival Transplantation better than dialysis (Y/N)*	*NOS score*
(2007)		(registry)	Scandiatransplant		years				LTx (grouped)	PD					
						nDM	1028	403					NR	Y	
Patel et al. (2008)	UK (Scotland)	Cohort Study	Single site	2002-2005	Median: 2.64	–	142	80	DTx	HD and PD	–	–	NR	Y	7
Pauly et al. (2009)	US and Canada	Cohort Study (registry)	2 dialysis sites in Canada and matched cohort from USRDS	1994-2006	Median: 3.77 – NHD<br><br>Median: 4.62 – DTx<br><br>Median: 4.30 – LTx	–	177	531 SCD	SCD	Home nocturnal HD	46.4 ± 11.8	46.9 ± 12.2	65% (WL) 57.8% (DTx)	N	9
							""	531 LTx	LTx				57.1% (LTx)	Y	
Heldal et al. (2010)	Norway	Cohort study (registry)	Norwegian Renal Registry	1990-2005	Max: 19	≥70 years	53	233 (36 pre-emptively)	DTx and LTx	HD and PD	Median: 73.4 (IQR: 69.5-82.0)	Median: 74.5 (IQR: 71.0-82.1)	81% (WL) 68% (Tx)	N – Start time of dialysis era: 1990-1994<br>Y – Start time of dialysis era: 2000-2005	9
Kumar et al. (2011)	UK (England)	Cohort study	Single site	2006-2009	Mean: 2.5 ± 1	Significant coronary disease*	88 (all patients had a PCI)	51 (all patients had a CABG)	DTx	NR	NR	NR	NR	Y	7
Bisigniano et al. (2012)	Argentina	Cohort study (registry)	SINTRA software (INCUCAI - National registry)	2005-2009	Max: 5	> 18 years	3647	1682	DTx	NR	47.88	48.14	NR	Y	9
De Lima et al. (2012)	Brazil	Cohort study	Single site	NR	Mean: 1.9 ± 1.3 (WL)<br>Mean: 2.1 ± 1.5 (Tx)<br><br>Max: 4	> 18 years	888	270	DTx	HD	54.8 ± 11.0	50.1 ± 10.0	62% (WL) 57% (Tx)	Y (high-risk patients)<br>N (low-risk patients)<br>Defined by the American Society of Transplantation	7
Bouaoun et al. (2013)	France	Cohort study (registry)	REIN	2002-2009	Max: 8 years	–	1706	2146	DTx and LTx	NR	50.2 ± 13	47.85 ± 14.1	61.5% (WL) 63.9% (Tx)	Y	9
Gill et al. (2013)	US	Cohort study (registry)	USRDS	1995-2007	Median: 2.67	≥65 years	14,396	11,072	SCD, LTx, and ECD	NR	NR	NR	63% (Overall)	Y (LTx > SCD > ECD)	8
						High risk**	""	3850	46% SCD; 31% ECD; 23% LD				65% (Overall)	Y	

Study Characteristics						Population type								Outcomes and quality	
Author and year	***Country***	***Study design***	***Data source***	***Inclusion period***	***Follow-up (years)***	***Population type***	***Waitlist group sample size***	***Transplant group sample size***	***Donor type***	***Dialysis type***	***Mean Age ± SD in years (range) – WL***	***Mean Age ± SD in years (range) – Tx***	***Male (%)***	***For Long-Term Survival Transplantation better than dialysis (Y/N)***	***NOS score***
						Intermediate risk**	“”	1397	46% SCD; 30% ECD; 24% LD				69% (Overall)	Y	
						Low risk**	“”	5825	45% SCD; 29% ECD; 25% LD				60% (Overall)	Y	
Schold et al. (2014)	US	Cohort study (registry)	SRTR	2003-2010	Median: 3.6<br>Max: 5	≥18 years	131,845	59,199	DTx	NR	NR	NR	60% (Overall)	Y<br>Y – centres with higher-than-expected outcomes<br>Y – centres with as-expected outcomes	9
Lloveras et al. (2015)	Spain	Cohort study (registry)	RMRC	1990-2010	Median: 3.2 years<br>Max: 21 years	–	823	823	ECD (≥65 years of age)	HD	61.7 ± 8.2	61.6 ± 7.8	64% (Overall)<br>64% (WL)<br>64% (Tx)	Y	9
Brar et al. (2016)	US	Cohort study (registry)	USRDS	2001-2007	Max: 8	Incident dialysis patients with PAD and <70 years	1843	2121 (grouped)	DTx and LTx	NR	Median: 40-59	Median: 40-59	68.6% (WL)<br>71.3% (Tx)		9
							“”	1328 DTx			Median: 40-59	Median: 40-59	70.9% (DTx)	N	
							“”	793 LTx			Median: 40-59	Median: 40-59	72% (LTx)	Y	
Cassuto et al. (2016)	US	Cohort study (registry)	UNOS	1994-2008	Max: 15 years	≥18 years of age with PAD	13647	4430 (671 pre-emptive)	SCD, LTx, and ECD (61.9% SCD; 21.7% LTx; 16.4% ECD)	NR	NR	55.1 ± 10.7	54.3% (Tx)<br>NR (WL)	Y (LTx > SCD > ECD)	9
Perez-Saez et al. (2016)	Spain	Cohort study (registry)	RMRC	1990-2013	Max: 25 (WL)<br>Max: 23 (Tx)	–	1651	389	DTx (≥75 years of age)	NR	–	66.9 ± 6.2	65.6% (Overall)<br>67.2% (WL)<br>58.8% (Tx)	Y (Overall)<br>N (KF-DM)<br>N (COPD)<br>N (aged ≥70 years)	9
Sorensen et al. (2016)	Denmark	Cohort study (registry)	DNR and the Scandiatransplant	1995-2011	Max: 22 years	–	825	2349 (of which 1535 were LTx)	DTx and LTx	NR	Median: 55-64	Median: 18-44	62% (WL)<br>62% (Tx)	Y (LTx > DTx)	9
Kaballo et al. (2018)	Ireland	Cohort study (registry)	National registry	2004-2013	Mean: 2.5	≤70 years	1157	990	DTx	NR	Median: 40-59	Median: 40-59	65% (WL)<br>63% (Tx)	Y	9
Legeai et al. (2018)	France	Cohort study (registry)	REIN	2002-2013	Mean: 2.0 ± 2.2	≥ 70 years	342	877 (160 pre-emptive)	SCD, LTx, and ECD (39 SCD;	NR	73.4 ± 2.9	73.0 ± 2.4	71.1% (WL)<br>67.8% (Tx)	N (risk with Tx had halved by 9 months compared to first 3	9

Study Characteristics						Population type								Outcomes and quality	
Author and year	*Country*	*Study design*	*Data source*	*Inclusion period*	*Follow-up (years)*	*Population type*	*Waitlist group sample size*	*Transplant group sample size*	*Donor type*	*Dialysis type*	*Mean Age ± SD in years (range) – WL*	*Mean Age ± SD in years (range) – Tx*	*Male (%)*	*For Long-Term Survival Transplantation better than dialysis (Y/N)*	*NOS score*
									28 LTx; 810 ECD)					months, the perioperative risk was still not offset by month 36)	
Clark et al. (2019)	US	Cohort study (registry)	USRDS	1995-2014	Mean: 6.5 ± 4.6	18-75 years	322,267	127,670	DTx	NR	NR	NR	NR	Y	7
Sawinski et al. (2019)	US	Cohort study (registry)	DaVita linkage with OPTN	2004-2014	Median: 1.8	≥18 years and HCV+	34,018	1117	DTx (HCV- and HCV+)	HD and PD	NR	NR	NR	Y (HCV- > HCV+)	7
Arcos et al. (2020)	Spain	Cohort study (registry)	RMRC	1990-2014	Median: 4<br>Max: 21	≥60 years	1373	1212 (Overall)	NR	HD and PD	–	Mean: 70.1	64.8% (WL)<br>67.9% (Tx)	Y	9
							“”	1084 (donors 60-79y)					61% (Tx)	Y – Global<br>Y – <70y<br>Y – ≥70y	
							“”	128 (donors ≥80y)					58.6% (Tx)	Y – Global<br>Y – <70y<br>N – ≥70y	
Fragale et al., 2020	Argentina	Cohort study	Multiple sites (n = 8) with linkage to SINTA	2006-2016	Median: 2.5<br>Max: 4.1	≥60 years	351	351	DTx	NR	66.66 ± 4.91	66.31 ± 4.59	61% (WL)<br>59% (Tx)	Y	9
Lenain et al., 2021	France	Cohort study (registry)	REIN	2005-2016	Median: 3.5	Adult patients	10646	10,646	DTx	NR	Median: 56.9 (IQR: 45.8-65.1)	Median: 55.3 (IQR: 44.5-64.0)	64% (WL)<br>64% (Tx)	Y	9
Hellemans et al., 2021	Belgium	Cohort study (registry)	ERA-EDTA linkage with Belgium renal registries	2000-2012	Max: 16	≥18 years	426	3382	SCD and ECD	HD and PD	Median: 56 (IQR: 47-63)	Median: 53 (IQR: 44-61)	60% (WL)<br>63% (Tx)	Y (SCD > ECD)	9

Abbreviations: ANZDATA = Australia And New Zealand Dialysis And Transplant Registry; BMI = Body Mass Index; CABG = Coronary Artery Bypass Graft; COPD = Chronic Obstructive Pulmonary Disease; CORR = Canadian Organ Replacement Register; DM = Diabetes Mellitus; DNR = The Danish National Register On Regular Dialysis And Transplantation; DTx = Deceased Donor Transplantation; Davita = National Dialysis Provider In 47 States; ECD = Extended Criteria Donor; ERA-EDTA = European Renal Association-European Dialysis And Transplant Association; HCV+ = Hepatitis C Virus Positive; HCV+ Donor = Hepatitis C Virus Donor; HCV– Donor = Hepatitis C Virus Negative Donor; HD = Haemodialysis; INCUCAI = Instituto Nacional Central Unico Coordinador De Ablacion E Implante; KF-DM = Kidney Failure caused by Diabetes Mellitus; KF-DM1 = Kidney Failure caused by Diabetes Mellitus Type 1; KF-GN = Kidney Failure caused by Glomerulonephritis; KF-HTN = Kidney Failure caused by Hypertension; KF-Hereditary = Kidney Failure caused by Hereditary Cause; LTx = Living Donor Transplantation; MORE = Multiple Organ Retrieval and Exchange Program (Regional Registry); nDM = No Diabetes Mellitus; nObese = Not Obese; NOS = Newcastle-Ottawa Scale; NR = Not Reported; PAD = Peripheral Arterial Disease; PCI = Percutaneous Coronary Intervention; PD = Peritoneal Dialysis; REIN = Renal Epidemiology And Information Network Registry; RMRC = Catalan Renal Registry; SCD = Standard Criteria Donor; SINTRA = Sistema Nacional De Información De Procuración Y Trasplante De La República Argentina; SRTR = Scientific Registry Of Transplant Recipients; Tx = Transplantation; UK = United Kingdom; UNOS = United Network For Organ Sharing; US = United States; USRDS = United States Renal Data System; WL = Waitlisted Dialysis Group

Note:

The studies are sorted by year and the first author's last name.
Text highlighted in red indicates group or subgroups of patients in which long-term morality risk, between those who underwent transplantation and those who remained waitlisted on dialysis, was not statistically different.

**Defined as >75% stenosis of one or more coronary vessels, >50% left main stem disease, or an equivocal lesion with flow limitation*

***High risk group = all patients with diabetes as either cause of kidney failure or as comorbid condition were considered high risk*
***Intermediate risk = if they had one or two of the following comorbid conditions: ischemic heart disease, congestive heart failure, cerebrovascular accident, peripheral vascular disease*
***Low risk = if patients had 0 cardiovascular comorbid conditions*

Supplementary Table D. Statistical analysis procedures adopted in studies and detailed outcomes

Author and year	*Country*	*Statistical analysis type*	Intention to treat approach	Start of analysis	End of analysis	*Adjusted model (Y/N)*	*Covariates in fully adjusted model*	*Detailed Outcomes*
Cantaluppi et al. (1977)	Italy	Survival was assessed using actuarial survival curves	NA	NR	NR	N	NA	No statistical difference between Tx and dialysis at any time interval
Golper et al. (1978)	USA	Survival analysis of the 2 patient groups was calculated by the life-table method. Patients were removed from the waitlist group upon transplantation and patients in the transplantation group were removed as alive if they had been on maintenance dialysis for 3 months following failure of their transplant.	NA	NR	NR	N	NA	No statistical difference between Tx and dialysis at any time interval
Fauchald et al. (1988)	Norway	Kaplan–Meier Estimator	NA	NR	NR	N	NA	>1 year survival was greater for Tx
Port et al. (1993)	USA	Time-dependent Cox regression model	Y	Listing on WL	Death, unavailable for follow-up or end of study period.	Y	Age, Sex, Race, Primary cause of ESRD, and time since waitlisting to transplantation	0–30-day survival was greater for Dx<br>RR: 2.43 ($P < 0.01$) – Overall<br>RR: 1.94 ($P > 0.05$) – Diabetes<br>RR: 1.58 ($P > 0.05$) – Glomerulonephritis<br>RR: 2.73 ($P > 0.05$) – Hypertension<br><br>>1 year survival was greater for Tx<br>RR: 0.36 ($P< 0.001$) – Overall<br>RR: 0.25 ($P < 0.01$) – Diabetes<br>RR: 1.16 ($P > 0.05$) – Glomerulonephritis<br>RR: 0.51 ($P > 0.05$) – Hypertension<br><br>Equal mortality risk = 117±28 days<br>Equal cumulative mortality = 325±91 days
Ojo et al. (1994)	USA	Time-dependent Cox regression model	Y	Listing on WL	Death, unavailable for follow-up or end of study period.	Y	Age, Sex, Race, Primary cause of ESRD, and time since waitlisting to transplantation	0–30-day survival was greater for Dx<br>RR: 3.3 ($P < 0.03$)<br><br>>1 year survival was greater for Tx<br>RR: 0.49 ($P< 0.03$)<br><br>Equal mortality risk = 112 days<br>Equal cumulative mortality = 378 days
Bonal et al. (1997)	Spain	Cox proportional hazard model with Epilog program. Relative risk estimates were determined with maximal likelihood method and chi-square test.	Y	NR	NR	Y	Age group, functional autonomy degree, and presence or absence of cardiovascular comorbidities (IHD, cardiomyopathy, cardiac arrythmia or stroke)	In patients 50–59-years Tx offered survival advantage.<br><br>HR: 0.51 ($P = 0.02$) – Overall<br>HR: 55-59 (ref) = 1<br>HR: 60-64 = 1.07 ($P = 0.75$)<br>HR: 65-70 = 1.87 ($P = 0.01$)<br><br>In patients 65-70 years no survival difference was observed between WL dialysis group and Tx
Segoloni et al. (1998)	Italy	Kaplan–Meier Estimator with Breslow and log-rank tests	NR	Transplant	NR	N	NA	Survival advantage greater with Tx compared to dialysis for the overall cohort and for >50 years (not statistically significant)
Wolfe et al. (1999)	USA	Time-dependent Cox regression model	Y	Listing on WL	Death, unavailable for follow-up or end of study	Y	Age, sex, race, cause of ESRD, year of placement on the waiting list, geographic region, and time from the	>18-month survival was greater for Tx<br>RR: 0.32 (0.30 to 0.35; $P < 0.001$) – Overall<br>RR: 0.24 (0.20 to 0.29; $P < 0.001$) – 20-39 year<br>RR: 0.33 (0.29 to 0.37; $P < 0.001$) – 40-59 year

Author and year	Country	Statistical analysis type	Intention to treat approach	Start of analysis	End of analysis	Adjusted model (Y/N)	Covariates in fully adjusted model	Detailed Outcomes
					period.		first treatment for ESRD to placement on the waiting list	RR: 0.39 (0.33 to 0.47; P < 0.001) – 60-74 year<br>RR: 0.27 (0.24 to 0.30; P < 0.001) – ESRD-DM<br>RR: 0.39 (0.31 to 0.48; P < 0.001) – ESRD-GN<br><br>Overall Equal mortality risk = 106 days<br>Equal cumulative mortality = 325±91 days
Medin et al. (2000)	Sweden	Kaplan–Meier Estimator and Cox proportional hazard model	Y	Listing on WL	Death, unavailable for follow-up or end of study period.	Y	Age	Long term survival advantage was greater with Tx (DTx or LTx) compared to dialysis. Most pronounced difference was between LTx and DTx rather than between DTx and dialysis.<br><br>RR of death:<br>DTx = 1 (ref)<br>LTx = 0.46 (0.27 to 0.78)<br>WL = 1.49 (1.12 to 1.99)
Rabbat et al. (2000)	Canada	Time-dependent Cox regression model	Y	Listing on WL	Death, unavailable for follow-up or end of study period.	Y	Age, race, gender, and time from start of ESRD therapy to wait-listing	Long term survival advantage was greater with Tx compared to dialysis. Survival advantage was most pronounced in patients with diabetes and glomerulonephritis as causes of ESRD.<br><br>0–30-day survival was greater for Dx<br>RR: 2.91 (1.34 to 6.32) – Overall<br>RR: 1.87 (0.44 to 7.89) – Diabetes<br>RR: 1.55 (0.91 to 11.71) – Glomerulonephritis<br>RR: 8.46 (0.87 to 82.54) – Hypertension<br>RR: 8.82 (0.91 to 85.67) – Hereditary<br>RR: 5.89 (1.26 to 27.58) – Other<br><br>>1 year survival was greater for Tx<br>RR: 0.25 (0.14 to 0.42) – Overall<br>RR: 0.38 (0.17 to 0.87) – Diabetes<br>RR: 0.13 (0.04 to 0.39) – Glomerulonephritis<br>RR: 0.56 (0.11 to 2.77) – Hypertension<br>RR: 0.45 (0.06 to 3.26) – Hereditary<br>RR: 0.22 (0.05 to 0.93) – Other
Kalo et al. (2001)	Hungary	Mortality data was analysed using the standardized mortality hazard function, the standardized survival function, the relative and absolute risk reduction of mortality, and the number of patients needed to treat by transplantation to avoid 1 death. Cox regression was then used to calculate mortality differences.	NR	NA	NA	Y	Age, sex, and length of ESRD before commencement of the study period	3-year survival was greater for Tx<br>RR: 0.723 (P = 0.06)
Straathof-Galema et al. (2001)	Netherlands	Kaplan–Meier Estimator	NR	Listing on WL or date of first dialysis therapy, whichever came last	Death, unavailable for follow-up or end of study period.	N	NA	No statistical difference between Tx and dialysis at any time interval
McDonald et al. (2002)	Australia; New Zealand	Time-dependent Cox regression model	Y	Listing on WL	Death, unavailable for follow-up or end of study	Y	Age, gender, race, and co-morbidities (diabetes, smoking, coronary artery disease, and peripheral	0–3-month survival was greater for Dx<br>HR: 2.0 (1.5 to 2.7; P< 0.001)<br>≥1-year survival was greater for Tx

Author and year	***Country***	***Statistical analysis type***	**Intention to treat approach**	**Start of analysis**	**End of analysis**	***Adjusted model (Y/N)***	***Covariates in fully adjusted model***	***Detailed Outcomes***
					period.		vascular disease)	HR: 0.19 (0.15 to 0.24; P< 0.001)
Brunkhorst et al. (2003)	Germany	Kaplan–Meier Estimator and Cox model	NR	Listing on WL	Death, graft loss or both	Y	Unclear but believed to be: Dialysis, serum cholesterol, mean, arterial blood pressure, number of antihypertensive drugs, serum calcium, serum phosphorus, and HbA1c	Long term survival advantage was greater with Tx compared to dialysis in patients with ESRD caused by T1DM.<br><br>RR: 0.46 (0.26 to 0.82; P = 0.009) - 10 year mortality risk
Glanton et al. (2003)	USA	Time-dependent (discrete) Cox regression model	Y	Listing on WL	Death, latest available follow-up or end of study period.	Y	Age, race, cause of ESRD, year of first dialysis, presence of congestive heart failure, and serum albumin levels	Long term survival advantage was greater with Tx (DTx or LTx) compared to dialysis. Magnitude of benefit of Tx over dialysis was similar for both obese and non-obese ESRD patients. Benefit of DTx did not apply to patients with BMI ≥41 kg/m2.<br><br>HR: 0.39 (0.33 to 0.47; P < 0.001) – DTx<br>HR: 0.23 (0.16 to 0.34; P < 0.001) – LTx<br>HR: 0.47 (0.17 to 1.25; P = 0.13) – BMI ≥ 41 kg/m^2<br>HR: 1.81 (1.55 to 2.11; P < 0.001) – ESRD-DM vs all other causes (CTx)<br>HR: 1.86 (1.60 to 2.18; P < 0.001) – ESRD-DM vs all other causes (LTx)<br>HR: 1.74 (1.42 to 2.12; P < 0.001) – PVD vs. absent (CTx)<br>HR: 1.70 (1.39 to 1.64; P < 0.001) – PVD vs. absent (LTx)<br>HR: 1.42 (1.22 to 1.65; P < 0.001) – CHF vs. absent (CTx)<br>HR: 1.40 (1.19 to 1.66; P < 0.001) – CHF vs. absent
Abbott et al. (2004)	USA	Time-dependent (discrete) Cox regression model	Y	Listing on WL	Death, unavailable for follow-up or end of study period.	Y	Model was adjusted for demographic variables (age, race, sex, year of listing) as well as a stepwise fit of all variables shown to be significantly associated with survival in previous studies. In addition, they also fitted access complications and claims for HCV at the time of listing.	Long term survival advantage was greater with DHCV+ Tx compared to dialysis on univariate analysis.<br><br>HR: 0.76 (0.60 to 0.96; P < 0.03)<br><br>Benefit was not seen in adjusted analysis because of significant confounding of allocation of DHCV+ kidneys.
Gibney et al. (2004)	USA	Kaplan–Meier Estimator and log-rank tests	NR	NA	NA	N	NA	Despite suboptimal outcomes in graft survival and frequent occurrence of early graft loss, for patients with SS there the long-term survival advantage was greater with DTx compared to dialysis.
Oniscu et al. (2004)	UK (Scotland)	Time-dependent Cox regression model	N	Listing on WL	Death, unavailable for follow-up or end of study period.	Y	Age, gender, social deprivation, primary renal disease, distance from patient's home to transplant centre, and time on dialysis pre-listing	Long term survival advantage was greater with Tx compared to dialysis.<br><br>0-30d survival was greater for Dx<br>RR: 4.91 (2.09 to 11.52; P> 0.05)<br>RR: 5.03 (1.43 to 17.73; P > 0.05) – Patients with comorbidity data (60%)<br><br>1 year survival was greater for Tx<br>RR: 0.35 (0.22 to 0.54; P< 0.05)<br>RR: 0.27 (0.14 to 0.52; P < 0.05) – Patients with comorbidity data

Author and year	***Country***	***Statistical analysis type***	**Intention to treat approach**	**Start of analysis**	**End of analysis**	***Adjusted model (Y/N)***	***Covariates in fully adjusted model***	***Detailed Outcomes***
								(60%)
Gill et al. (2005)	USA	Time-dependent Cox regression model	Y	Listing on WL	Death or end up study follow-up	Y	Age, gender, race, cause of end-stage kidney disease, comorbid conditions defined at the time of dialysis initiation (ischemic heart disease, congestive heart failure, stroke, peripheral vascular disease), duration of dialysis exposure prior to transplantation, and year of placement on the transplant waiting list.	Long term survival advantage was greater with Tx compared to dialysis.<br><br>RR >1-year after transplantation versus remaining on dialysis:<br>0.49 – pre-emptive Tx<br>0.43 – 1 year waiting time<br>0.38 – 2 year waiting time<br>0.34 – 3 year waiting time
Merion et al. (2005)	USA	Time-dependent Cox regression model	Y	Listing on WL or date of first dialysis therapy, whichever came first	survival time at risk was censored at the time of living-donor transplantation, wait listing for another organ, or end of study.	Y	Age, sex, race, ethnicity, blood type, ESRD cause, panel reactive antibody values, dialysis modality, comorbid conditions present at wait-listing year, donation service area for the organ procurement organization (OPO) of candidate registration, and time from dialysis to wait-listing.	Long term survival advantage was greater with Tx (SCD or ECD) compared to dialysis.<br><br>3 year:<br>RR: 0.28 (0.27 to 0.30; P < 0.001) – SCD<br>RR: 0.40 (0.37 to 0.44; P < 0.001) – ECD<br><br>Patients with diabetes, hypertensive nephrosclerosis, and older candidates had the largest survival benefit.<br><br>Cumulative survival with ECD took 3.5 years post-transplantation to equal that of standard-therapy patients (SCD and wait-listed dialysis).
Oniscu et al. (2005)	UK (Scotland)	Time-dependent Cox regression model (adjusted for demographic variables in model 1 and for demographic and co-morbidity variables in model 2). Also performed subsequent analysis in which censoring was done at graft failure and was considered towards the follow-up in the dialysis group	Y	Listing on WL	Death, latest available follow-up or end of study period.	Y	Age, gender, primary renal disease, social deprivation, time since waitlisting (model 1), and comorbidity (model 2)	Long term survival advantage was greater with Tx compared to dialysis. Benefit was present in all patients undergoing transplantation, irrespective of their age group or primary renal disease.<br><br>0-30 day<br>RR: 1.35 (0.63 to 2.86) – No comorbidity adjustment<br>RR: 0.91 (0.22 to3.70) – Comorbidity adjusted<br>RR: 1.82 (0.22 to 14.80) – Multisystem disease<br>RR: 1.28 (0.17 to 9.83) – Diabetes<br>RR: 2.38 (0.27 to 20.84) – >65 yr<br><br>>1 year:<br>RR: 0.32 (0.25 to 0.40; P < 0.001) – No comorbidity adjustment<br>RR: 0.28 (0.20 to 0.39; P < 0.001) – Comorbidity adjusted<br>RR: 0.16 (0.07 to 0.39; P < 0.05) – Glomerulonephritis<br>RR: 0.28 (0.13 to 0.61; P < 0.05) – Interstitial nephritis<br>RR: 0.13 (0.04 to 0.38; P < 0.05) – Multisystem disease<br>RR: 0.33 (0.15 to 0.74; P < 0.05) – Diabetes<br>RR: 0.34 (0.14 to 0.83; P < 0.05) – >65 yr<br><br>1.5 year:

Author and year	*Country*	*Statistical analysis type*	Intention to treat approach	Start of analysis	End of analysis	*Adjusted model (Y/N)*	*Covariates in fully adjusted model*	*Detailed Outcomes*
								RR: 0.18 (0.08 to 0.42; P < 0.001)
Snyder et al. (2006)	USA	Time-dependent Cox regression model in patients with and without diabetes separately with PAD	Y	NR	NR	Y	Age, gender, race, ethnicity, BMI, primary ESRD cause, baseline comorbidities and time on RRT	Long term survival advantage was greater with Tx compared to dialysis.<br><br>3 year:<br>RR: 0.36 (0.31 to 0.41; P < 0.001) – DM PAD+<br>RR: 0.57 (0.52 to 0.62; P < 0.001) – DM PAD-<br>RR: 0.47 (0.40 to 0.56; P < 0.001) – nDM PAD+<br>RR: 0.73 (0.68 to 0.79; P < 0.001) – nDM PAD-
Gill et al. (2007)	USA	Unadjusted death rates were determined in 3-month intervals during periods of waitlisting, allograft function, and after allograft failure.	NA	NA	NA	N	NA	Long term survival advantage was greater with Tx compared to dialysis.
Rao et al. (2007)	USA	Time-dependent Cox regression model	Y	Listing on WL or date of first dialysis therapy, whichever came first	NR	Y	Age, sex, race, ethnicity, panel, reactive antibody level, diagnosis, dialysis modality, donation service area, and time from initiation of dialysis until first placement on the waiting list	Long term survival advantage was greater with Tx compared to dialysis. Benefit was most striking for patients with ESRD caused by diabetes and/or hypertension.<br><br>HR: 0.59 (0.53 to 0.65; P < 0.001) – All (70+ yr)<br>HR: 0.58 (0.52 to 0.65; P < 0.001) – HR: Age 70-74 yr<br>HR: 0.67 (0.53 to 0.86; P < 0.05) – Age 75+ yr<br><br>HR: 0.75 (0.65 to 0.86; P < 0.001) - ECD<br>HR: 0.43 (0.33 to 0.57; P < 0.001) – LTx<br><br>HR: 0.89 (0.64 to 1.22; ns) – Glomerulonephritis<br>HR: 0.53 (0.41 to 0.68; P < 0.001) – Diabetes<br>HR: 0.56 (0.45 to 0.68; P < 0.001) – Hypertension<br>HR: 0.49 (0.39 to 0.61; P < 0.05) – Other ESRD
Savoye et al. (2007)	France	Kaplan–Meier Estimator and log-rank tests / Time-dependent Cox regression model	Y	NR	NR	Y	Age, peak panel reactive antibody level, initial cause of ESRD, blood group, and time on dialysis before registration	Long term survival advantage was greater with Tx (SCD or ECD) compared to dialysis.<br><br>Adjusted HR of death for remaining on dialysis vs receiving a transplant:<br>HR: 2.59 (2.08 to 3.21; P < 0.001) – Overall (ECD and SCD)<br>HR: 3.96 (2.84 to 5.51; P < 0.001) – Overall (SCD)<br>HR: 2.25 (1.80 to 2.81; P < 0.001) – Overall (ECD)
Sorensen et al. (2007)	Denmark	Cox proportional hazard regression model	Y	Listing on WL	NR	Y	Age, sex, diabetes, waiting list status, transplantation, and time period	Long term survival advantage was greater with Tx (DTx or LTx) compared to dialysis.<br><br>HR: 0.21 (0.13 to 0.34; P < 0.001)
Patel et al. (2008)	UK (Scotland)	Kaplan–Meier Estimator and log-rank tests / Cox proportional hazard regression model	NR	NR	NR	N	NA	Long term survival advantage was greater with Tx (DTx or LTx) compared to dialysis.<br><br>Mean survival Tx = 4.5 ± 0.6 years<br>Mean survival WL = 4.1 ± 1.4 years
Pauly et al. (2009)	USA and Canada	Kaplan–Meier Estimator and log-rank tests / Cox proportional hazard regression model	NR	Date of first treatment (either Nocturnal	Death or end up study follow-up	Y	Age at NHD start or transplantation, gender, history of ischaemic heart disease/peripheral vascular disease/cancer, study year	No difference in the adjusted survival of ESRD patients treated with nocturnal HD and DTx. Conversely, recipients of LTX demonstrated the best survival benefit.<br><br>HR: 0.87 (0.50 to 1.51; P = 0.61) – SCD

Author and year	*Country*	*Statistical analysis type*	Intention to treat approach	Start of analysis	End of analysis	*Adjusted model (Y/N)*	*Covariates in fully adjusted model*	*Detailed Outcomes*
				HD/DTx/LTx)			and duration of conventional dialysis treatment prior to treatment with treatment modality of interest.	HR: 0.51 (0.28 to 0.91; P = 0.02) – LTx
Heldal et al. (2010)	Norway	Kaplan–Meier Estimator / Time-dependent Cox regression model	Y	Listing on WL or start of dialysis (whichever was latest)	Death or end of study period.	Y	Age, sex, primary kidney disease, type of centre (university vs not university hospital), time on dialysis before waitlisting, and dialysis modality.	Long term survival advantage in elderly patients was greater with Tx compared to dialysis.<br><br>HR: 0.78 (0.52 to 1.18, P = 0.25) – Overall<br><br>Start time of dialysis era:<br>HR: 1.01 (0.58 to 1.75) – 1990-1994<br>HR: 0.40 (0.19 to 0.83; P = 0.01) – 2000-2005
Kumar et al. (2011)	UK (England)	Kaplan–Meier Estimator and log rank test	NR	NA	NA	N	NA	Long term survival advantage was greater with Tx compared to dialysis.<br><br>Survival at 1 and 3 years after angiogram was 100% and 97.2% and for dialysis patients it was 94.9% and 80.7% at 1 and 3 years, respectively.
Bisigniano et al. (2012)	Argentina	Time-dependent Cox regression model	NR	Listing on WL	Death, latest available follow-up or end of study period.	Y	Age, gender, ischemia (< or >24hr), time on dialysis (< or >7yrs), primary cause of ESRD, and donor cause of death	0–30 days survival was greater for Dx<br>HR: 4.18 (2.88 to 6.06; P < 0.001)<br><br>>1 year survival was greater for Tx<br>HR: 0.19 (0.12 to 0.29; P < 0.001)
De Lima et al. (2012)	Brazil	Kaplan–Meier Estimator and log rank test		–	–	N	NA	Benefit of transplantation was mainly restricted to the elderly and to patients with diabetes and/or CVD, defined by the American Society of Transplantation as high-risk patients. Low-risk patients did well either on dialysis or Tx so that no discernible advantage.
Bouaoun et al. (2013)	France	Used Lexis diagrams to analyse two-time scales: time since ESRD onset and time since waitlisting, both until transplantation or end of follow-up. Crude death rates were computed for each period by dividing the number of deaths by the time spent in each status and expressed per 100 person-years.	NR	NA	NA	N	NA	Long term survival advantage in patients was greater with Tx compared to dialysis.
Gill et al. (2013)	USA	Time-dependent Cox regression model	Y	Listing on WL	Death, delisting, and end of study period	Y	Age, gender, race, primary cause of ESRD, and year of wait listing	Long term survival advantage in patients was greater with Tx (DTx, LTx, or ECD) compared to dialysis.<br><br>In patients with low and intermediate cardiovascular risk, LTx transplantation was associated with almost an immediate survival advantage.
Schold et al. (2014)	USA	Time-dependent Cox regression model	Y	Listing on WL	Censored at time of living transplantation or last follow-up	Y	Age, BMI, race, sex, insurance status, panel reactive antibody level, educational attainment, active versus inactive status, time on dialysis, and primary cause of ESRD.	Long term survival advantage in patients was greater with Tx compared to dialysis. This was found to be true regardless of treatment centre performance.<br><br>HR: 0.32 (0.31 to 0.32) – Overall<br>HR: 0.23 (0.20 to 0.25) – centres with higher-than-expected outcomes<br>HR: 0.30 (0.30 to 0.31) – centres with as-expected outcomes

Author and year	*Country*	*Statistical analysis type*	Intention to treat approach	Start of analysis	End of analysis	*Adjusted model (Y/N)*	*Covariates in fully adjusted model*	*Detailed Outcomes*
Lloveras et al. (2015)	Spain	Kaplan–Meier Estimator and log-rank tests / Cox proportional hazard regression model (clustering by pairs)	NR	Date of transplant (Tx group) or date of equal dialysis time (for WL group)	Death, unavailable for follow-up or end of study period.	Y	Age, sex, primary cause of ESRD, HCV+ status, donor age, chronic liver and respiratory diseases, malignant tumours, and DM	Long term survival advantage in patients was greater with Tx (donor ≥65y) compared to dialysis.<br><br>HR of remaining on dialysis vs receiving a Tx:<br>HR: 2.66 (2.21 to 3.20; P < 0.001)
Brar et al. (2016)	USA	Time-dependent Cox regression model	Y	Listing on WL	Death, latest available follow-up or end of study period.	Y	Hypercholesterolemia, CVA, IHD, HTN, DM, gender, history of amputation, and age at listing, time on dialysis from WL to transplantation.	Long term survival advantage in PAD patients was greater with LTx compared to dialysis. DTx did not offer a survival advantage over dialysis for PAD patients.<br><br>≤ 100 days<br>RR: 2.36 (1.67 to 3.32; P < 0.001) – CTx<br>RR: 0.93 (0.50 to 1.71; P = 0.81) – LTx<br><br>>1 year<br>RR: 1.17 (0.84 to 1.64; P = 0.36) – DTx<br>RR: 0.35 (0.22 to 0.57; P < 0.001) – LTx
Cassuto et al. (2016)	USA	Kaplan–Meier Estimator and log-rank tests / Time-dependent (discrete) Cox regression model		Listing on WL	Death, latest available follow-up or end of study period.	Y	Age, race, gender, dialysis status at time of transplant, primary cause of ESRD, CAD, and PAD. Donor characteristics included age, CIT, and donor quality.	Long term survival advantage in PAD patients was greater with Tx (SCD, LTx, or ECD) compared to dialysis. LTx was associated with the greatest post-transplant survival benefit.<br><br>3-year survival:<br>HR: 0.89 (0.74 to 0.99; p = 0.04 – ECD<br>HR: 0.52 (0.47 to 0.58; p < 0.01) – SCD<br>HR: 0.31 (0.26 to 0.37; p < 0.01) – LTx<br><br>5-year survival:<br>HR: 0.469 (0.435-0.504; P < 0.001)
Perez-Saez et al. (2016)	Spain	Time-dependent Cox regression model	Y	Listing on WL	Death	Y	Age, sex, waitlist period (before or after 2000), primary cause of ESRD, having at least one of five cardiovascular comorbidities (IHD, cardiac failure, cardiac conduction disorders, CVA, PVD), having a chronic respiratory disease, and time from first treatment for ESRD to placement on the waiting list.	Long term survival advantage in patients was greater with Tx (donor ≥75y) compared to dialysis.<br><br>HR: 0.44 (0.32 to 0.61; P < 0.001) – Overall<br>HR: 0.17 (0.06 to 0.48; P < 0.001) – <65 years<br>HR: 0.56 (0.34 to 0.92; P = 0.02) – 65-69 years<br>HR: 0.81 (0.52 to 1.28; P = 0.39) – ≥70 years<br>HR: 0.46 (0.32 to 0.64; P < 0.001) – non-diabetic cause of ESRD<br>HR: 0.33 (0.09 to 1.14; P = 0.08) – ESRD-DM<br>HR: 0.53 (0.36 to 0.78; P = 0.001) – No cardiovascular co-morbidities<br>HR: 0.31 (0.16 to 0.60; P = 0.001) – Cardiovascular co-morbidities<br>HR: 0.46 (0.33 to 0.66; P < 0.001) – No DM<br>HR: 0.35 (0.14 to 0.88; P = 0.03) – DM<br>HR: 0.44 (0.31 to 0.63; P = 0.001) – No COPD<br>HR: 0.45 (0.17 to 1.24; P = 0.001) – COPD<br><br>Survival benefit lost its significance in recipients whose ESRD cause was diabetes mellitus (HR 0.33, 95% CI 0.09–1.15), in recipients with

Author and year	*Country*	*Statistical analysis type*	Intention to treat approach	Start of analysis	End of analysis	*Adjusted model (Y/N)*	*Covariates in fully adjusted model*	*Detailed Outcomes*
								chronic obstructive pulmonary disease (HR 0.46, 95% CI 0.17 to 1.27) and in those aged ≥70 years (HR 0.82, 95% CI 0.52 to 1.28).
Sorensen et al. (2016)	Denmark	Time-dependent Cox regression model	Y	Listing on WL	NR	Y	Age, sex, renal diagnosis, time on dialysis before entering the WL, and CCI	Long term survival advantage in elderly patients and those with high co-morbidity was greater with Tx (DTx or LTx) compared to dialysis.<br><br>HR: 0.3 (0.23 to 0.39) – LTx<br>HR: 0.38 (0.32 to 0.45) – DTx
Kaballo et al. (2018)	Ireland	Time-dependent Cox regression model	Y	Listing on WL	Death, latest available follow-up or end of study period.	Y	Age, sex, cause of ESRD, year of placement on the waiting list, and time from first ESRD to placement on the waiting list.	Long term survival advantage in patients was greater with Tx compared to dialysis.<br><br>5-year mortality risk:<br>RR: 0.53 (0.37 to 0.77; P = 0.001)
Legeai et al. (2018)	France	Time-dependent Cox regression model	NR	Listing on WL	Death, unavailable for follow-up or end of study period.	Y	Age, gender, number of cardiovascular comorbidities, mobility status, diabetes , and time on dialysis at inclusion.	Although risk with Tx had halved by 9 months compared to first 3 months, the perioperative risk was still not offset by month 36. Transplantation did not appear to provide statistical survival benefit for patients without cardiovascular comorbidity or diabetes.<br><br>HR: 3.0 (1.9 to 4.8; P < 0.001) – 0-3 months<br>HR: 0.5 (0.3 to 1.0; P = 0.05) – 24-36 months<br>HR: 0.9 (0.5 to 1.6; P = 0.65) – >3 years
Clark et al. (2019)	USA	Time-dependent Cox regression model	Y	Listing on WL	Death, latest available follow-up or end of study period.	Y	Age at waitlisting, sex, race, cause of ESKD, BMI, year of waitlisting, comorbid conditions, insurance type, and employment status	Long term survival advantage in patients was greater with Tx compared to dialysis.<br><br>1-year:<br>HR: 0.40 (0.39 to 0.41; P < 0.001)
Sawinski et al. (2019)	USA	Time-dependent Cox regression model	Y	Listing on WL	Death, delisting, and end of study period	Y	Age, sex, race, diabetes, CVD, severe liver disease, insurance, income, panel-reactive antibody, dialysis vintage, BMI, dialysis modality, year of waitlisting interacted with time, haemoglobin level, platelet count, and albumin level.	Long term survival advantage in patients was greater with Tx (HCV-ve or HCV+ve donor) compared to dialysis.<br><br>Survival benefit was achieved at 9 months and 2 years for HCV-ve and HCV+ve donor Tx, respectively.<br><br>3 years:<br>HR: 0.42 (0.27 to 0.63) – (seropositive and seronegative donors)<br>HR: 0.42 (0.25 to 0.72) – (seronegative donors)<br>HR: 0.52 (0.30 to 0.93) – (seropositive donors)
Arcos et al. (2020)	Spain	Time-dependent Cox regression model	Y	Listing on WL	Death, unavailable for follow-up or end of study period.	Y	Age, gender, period of time (only for donors between 60 and 79 y), ESRD-DM, having at least 1 of 5 cardiovascular comorbidities (IHD, cardiac failure, cardiac conduction disorders, CVA and PVD), and chronic respiratory disease.	>1 year survival was greater for Tx<br>HR: 0.46 (0.39-0.55; P < 0.001)<br><br>HR of Tx vs dialysis on WL at 12m (donors 60-79yr):<br>HR: 0.50 (0.44 to 0.58; P = 0.02) – Global<br>HR: 0.45 (0.38 to 0.52; P < 0.001) – <70y<br>HR: 0.68 (0.51 to 0.90; P < 0.001) – ≥70y<br>HR: 0.51 (0.42 to 0.62; P < 0.001) – No cardiovascular<br>HR: 0.49 (0.39 to 0.61; P < 0.001) – Any Cardiovascular<br>HR: 0.47 (0.40 to 0.55; P < 0.001) – No diabetes as PRD<br>HR: 0.67 (0.47 to 0.94; P < 0.001) – Diabetes as PRD<br><br>Risk of Tx vs dialysis on WL at 12m (donors ≥80yr):<br>HR: 0.54 (0.38 to 0.77; P = 0.001) – Global

Author and year	***Country***	***Statistical analysis type***	**Intention to treat approach**	**Start of analysis**	**End of analysis**	***Adjusted model (Y/N)***	***Covariates in fully adjusted model***	***Detailed Outcomes***
								HR: 0.48 (0.30 to 0.76; P = 0.002) – <70y<br>HR: 0.68 (0.39 to 1.16; P = 0.15) – ≥70y<br>HR: 0.54 (0.35 to 0.83; P = 0.005) – No cardiovascular<br>HR: 0.51 (0.28 to 0.91; P = 0.02) – Any Cardiovascular<br>HR: 0.56 (0.38 to 0.82; P = 0.003) – No diabetes as PRD<br>HR: 0.43 (0.17 to 1.09; P = 0.08) – Diabetes as PRD
Fragale et al., 2020	Argentina	Kaplan–Meier Estimator and log-rank tests / Cox proportional hazard regression model	Y	NR	NR	NR	NR	Long term survival advantage in patients older than 60 was greater with Tx compared to dialysis. Benefit was independent of degree of comorbidities present at inclusion on the WL.<br><br>HR: 0.35 (0.27 to 0.45; P < 0.001)<br>HR: 1.62 (1.09 to 2.41; P < 0.02) – CCI ≥ 3 versus CCI score ≤3
Lenain et al., 2020	France	Time -dependent propensity score matching (using nearest neighbour calliper matching). To emulate a 1:1 randomized trial, they performed random matching without replacement. They then obtained patient survival was through Kaplan-Meier estimator, computed the number needed to treat as the inverse of the absolute reduction risk, and finally they computed a proportional hazard model in the matched sub-cohort.	Y	Listing on WL or date of first dialysis therapy if patients entered WL without prior dialysis	Death, unavailable for follow-up or end of study period.	Y	NR	Long term survival advantage in patients was greater with Tx compared to dialysis.<br><br>For every 13 transplantations, one life was saved.
Hellemans et al., 2021	Belgium	Used Cox regression to calculate mortality during the median waiting time, with waiting time for both the transplanted and non-transplanted patients based on a competing risk analysis. They added the result to the 3-year mortality risk after transplantation and compared that outcome to an equal amount of time (median waiting time of 3 years) in the dialysis control group.	Y	Date of active waitlisted dialysis	Death, unavailable for follow-up or end of study period.	Y	Age (20-44, 45-64, ≥65), sex, and ESRD-DM	Long term survival advantage in patients was greater with Tx (SCD or ECD) compared to dialysis.<br><br>Although for elderly patients ECD for were associated with a minimal benefit.

Supplementary Table E. Leave-one-out meta-analysis for long term all-cause mortality for transplantation versus dialysis.

Author, year	Estimate	Z value	P value	95% CI	I^2 (%)	Q	Q (P value)
Fragale et al. 2020	0.4644	-8.45	<0.001	0.3887 to 0.5548	95.5	399.31	<0.001
McDonald et al. 2002	0.4793	-9.31	<0.001	0.4106 to 0.5596	93.8	290.07	<0.001
De Lima et al. 2012 (Low Risk)	0.4364	-9.83	<0.001	0.3699 to 0.5149	95.4	390.57	<0.001
De Lima et al. 2012 (High Risk)	0.4435	-9.41	<0.001	0.3745 to 0.5253	95.4	394.40	<0.001
De Lima et al. 2012 (Low Risk and High Risk)	0.4250	-9.88	<0.001	0.3586 to 0.5037	95.5	380.40	<0.001
Rabbat et al. 2000	0.4652	-8.92	<0.001	0.3932 to 0.5505	95.5	401.54	<0.001
Sorensen et al. 2016 (DTx)	0.4608	-8.68	<0.001	0.3869 to 0.5489	95.5	403.95	<0.001
Sorensen et al. 2016 (LTx)	0.4662	-8.75	<0.001	0.3930 to 0.5532	95.5	399.46	<0.001
Sorensen et al. 2016 (DTx and LTx)	0.4746	-8.03	<0.001	0.3956 to 0.5693	95.7	398.84	<0.001
Lenain et al. 2021	0.4746	-8.03	<0.001	0.3956 to 0.5693	95.7	398.84	<0.001
Brunkhorst et al. 2003	0.4540	-9.12	<0.001	0.3831 to 0.5379	95.5	404.23	<0.001
Kalo et al. 2001	0.4451	-9.43	<0.001	0.3762 to 0.5266	95.5	399.80	<0.001
Kaballo et al. 2018	0.4499	-9.10	<0.001	0.3788 to 0.5344	95.5	399.84	<0.001
Segoloni et al. 1998	0.4347	-9.83	<0.001	0.3681 to 0.5133	95.3	385.56	<0.001
Straathof-Galema et al. 2001	0.4479	-9.48	<0.001	0.3794 to 0.5288	95.5	401.98	<0.001
Fauchald et al. 1988	0.4674	-8.80	<0.001	0.3946 to 0.5536	95.5	399.63	<0.001
Heldal et al. 2010	0.4462	-9.45	<0.001	0.3775 to 0.5275	95.5	401.20	<0.001
Arcos et al. 2020	0.4569	-8.37	<0.001	0.3804 to 0.5488	95.5	398.81	<0.001
Medin et al. 2000	0.4433	-9.41	<0.001	0.3742 to 0.5251	95.4	392.89	<0.001
Oniscu et al. 2005	0.4752	-8.72	<0.001	0.4020 to 0.5617	95.4	389.93	<0.001
Patel et al. 2008	0.4511	-9.39	<0.001	0.3820 to 0.5326	95.5	403.74	<0.001
Clark et al. 2019	0.4713	-6.39	<0.001	0.3741 to 0.5936	95.0	363.13	<0.001

Supplementary Table F. Meta-regression analysis for potential confounders

Parameter	Studies	Test of Moderators	R^2 (%)	β-Estimate	SE	Z value	P value	95% CI
Mean Age	12	0.3685 (P = 0.54)	0	-0.0071	0.0117	-0.6071	0.54	-0.0300 to 0.0158
Maximum duration of follow-up	14	0.6496 (P = 0.40)	0	-0.0126	0.0151	-0.8334	0.40	-0.0421 to 0.0170
Median period of data collection	15	0.0736 (P = 0.79)	3.16	0.0028	0.0103	0.2713	0.79	-0.0174 to 0.0230
